# The Human Lung Glycome Reveals Novel Glycan Ligands for Influenza A Virus

**DOI:** 10.1038/s41598-020-62074-z

**Published:** 2020-03-24

**Authors:** Nan Jia, Lauren Byrd-Leotis, Yasuyuki Matsumoto, Chao Gao, Alexander N. Wein, Jenna L. Lobby, Jacob E. Kohlmeier, David A. Steinhauer, Richard D. Cummings

**Affiliations:** 1 Beth Israel Deaconess Medical Center, Department of Surgery and Harvard Medical School Center for Glycoscience, Harvard Medical School, Boston, MA USA; 2 Department of Microbiology and Immunology, Emory University School of Medicine, Atlanta, GA USA; 3 Emory-UGA Center of Excellence of Influenza Research and Surveillance, (CEIRS), Atlanta, GA USA

**Keywords:** Glycoconjugates, Glycobiology, Influenza virus

## Abstract

Glycans within human lungs are recognized by many pathogens such as influenza A virus (IAV), yet little is known about their structures. Here we present the first analysis of the N- and O- and glycosphingolipid-glycans from total human lungs, along with histological analyses of IAV binding. The N-glycome of human lung contains extremely large complex-type N-glycans with linear poly-N-acetyllactosamine (PL) [-3Galβ1–4GlcNAcβ1-]_n_ extensions, which are predominantly terminated in α2,3-linked sialic acid. By contrast, smaller N-glycans lack PL and are enriched in α2,6-linked sialic acids. In addition, we observed large glycosphingolipid (GSL)-glycans, which also consists of linear PL, terminating in mainly α2,3-linked sialic acid. Histological staining revealed that IAV binds to sialylated and non-sialylated glycans and binding is not concordant with respect to binding by sialic acid-specific lectins. These results extend our understanding of the types of glycans that may serve as binding sites for human lung pathogens.

## Introduction

Glycosylation represents the major post-translational modification of cellular proteins^1^, and glycoproteins along with glycolipids are abundantly displayed on the plasma membranes of cells and in secretions^2^. Complex glycoconjugates regulate numerous biological processes such as quality control of protein folding, modulation of gene transcription, cellular signaling and adhesion, and trafficking of all immune cells and platelets^3–5^. The diversity of different human glycans represents the total human glycome, which is vast and undefined, yet anticipated to represent many hundreds of thousands of different structures^6^. Each glycan structure is genetically dictated through specific gene expression of so-called ‘glycogenes’, which overall number over 500 in the human genome^7^, representing 1–2% of the genome. However, much of our knowledge of the human glycome is confined to free glycans and those expressed on blood cells and plasma glycoproteins and antibodies^8–11^. While studies on glycomes of individual cell types, cultured cells and human surgical biopsies^12–15^ have advanced our understanding of the potential glycans in the human glycome, few studies have globally analyzed total human tissues or organs.

It is important to define human glycans not only for their roles in systems biology, but to also discern the potential attachment sites and ligands for many infectious organisms and viruses. Human lungs are frequently subjected to infection by a variety of pathogens including viruses and bacteria^16–18^. Influenza A virus (IAV) receptor recognition is one of the most intensively studied examples of pathogen-host interaction, as glycans expressed on the surface of human airway are essential in shaping the initiation of infection by IAV. It has been commonly believed that the IAV envelope protein, hemagglutinin, recognizes glycans with a terminal sialic acid in either α2,3- or α2,6-linkage and such recognition is required for infection. As a general paradigm, α2,6-sialylated structures are preferentially recognized by human IAVs, whereas avian strains preferentially bind α2,3-sialylated glycans^19,20^.

Despite much effort to understand the initial virus-host recognition event, little is known about the natural repertoire of glycans present in the human respiratory system. Current understanding of the human lung glycome remains at the tissue level with limited information obtained from lectin staining and mass spectrometric analyses of surgical biopsies from human lungs^21–23^. It is important to understand the landscape of glycosylation at the organ level, as an organ represents a full collection of all pulmonary cell types whereas biopsy specimens only focus on a small area of lung parenchyma, which may not contain all glycan structures found in a lung. Our recent studies using shotgun glycomics of total N-glycans of a human lung challenge the paradigm of sialic acid recognition, as we discovered many IAVs recognize phosphorylated glycans^24^. Thus, it is even more important to gain a fuller understanding of the complexity of the human lung glycome to assess the structures and functions of glycans in lung biology.

Here we present our analyses of the human lung glycome using mass spectrometry (MS), with complementary information generated by Western blot and histochemistry staining with anti-glycan antibodies, lectins and IAVs. We focused on the analysis of N- and O-glycans released from glycoproteins as well as glycosphingolipid (GSL)-derived glycans that were extracted from a perfused lung of a healthy young adult, the results of which are compared to a lung from a second donor. To advance our understanding of minor glycan species, we established a method to characterize phosphorylated glycans. The present work provides a comprehensive mapping of the human lung glycome and represents the first of its kind to dissect the human glycome at the organ level. This work represents a major step in the goals of the Human Glycome Project, which aims to define the structures and functions of human glycoconjugates (www.human-glycome.org).

## Results

### Western blot reveals the expression of sialylated and fucosylated glycans in human lung

**Figure 1:**
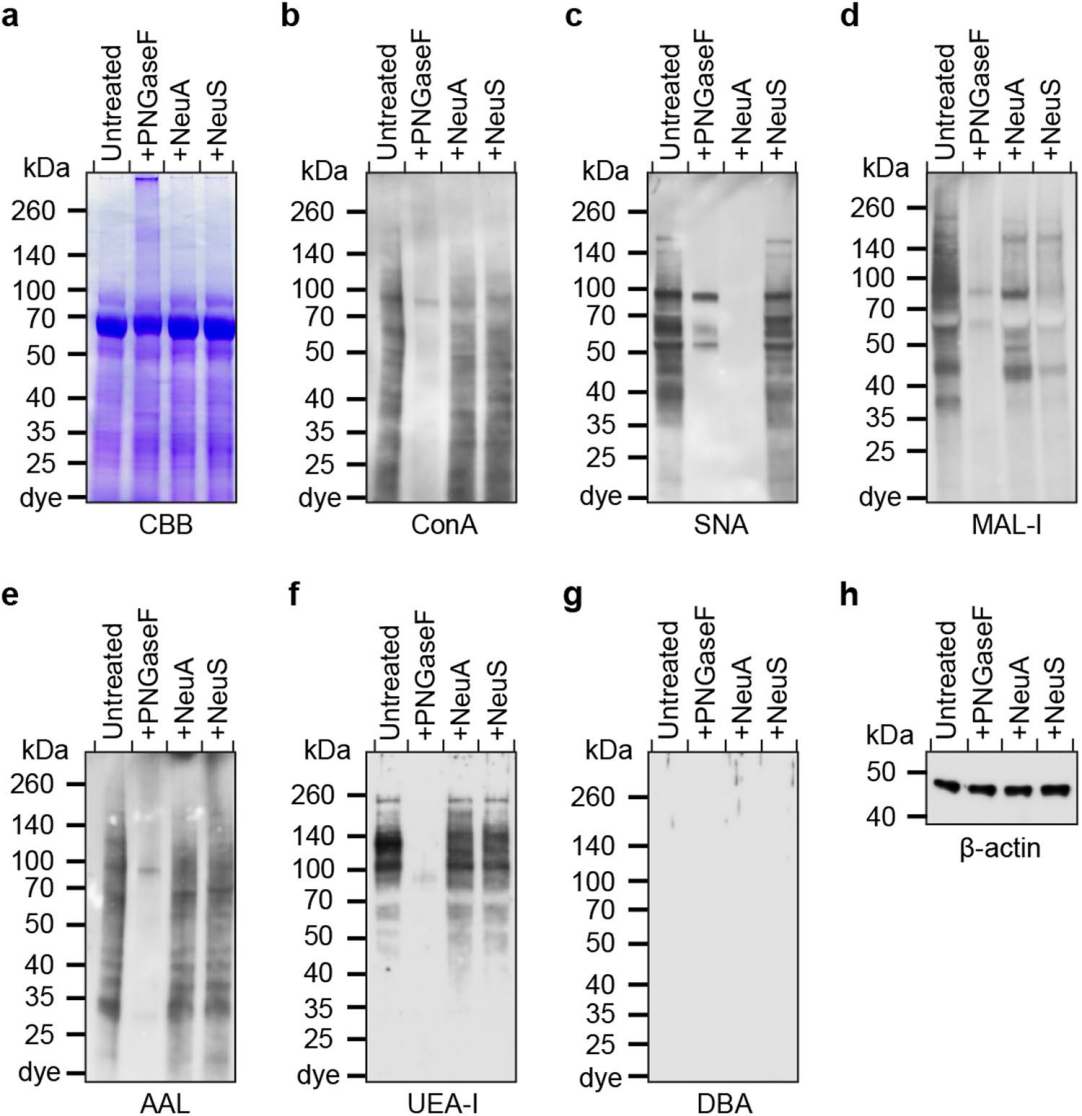
Western blot of human lung. Tissue homogenate in a human lung were treated with PNGase F (PNGaseF), Neuraminidase A (NeuA), or Neuraminidase S (NeuS), and separated by SDS-PAGE. The gel was stained with Coomassie Brilliant Blue solution (**a**), or analyzed by Western blot using ConA (**b**), SNA (**c**), MAL-I (**d**), AAL (**e**), UEA-I (**f**), and DBA (**g**). β-actin antibody staining was used as an internal control (**h**).

Human lungs used for analyses were perfused upon collection and devoid of blood cells and soluble mucus. We interrogated the glycome of human lung glycoproteins by probing for specific glycan epitopes in a lung homogenate using lectins and antibodies (Fig. 1a–h; Supplementary Fig. 1a–f). Staining with the lectin concanavalin A (ConA)^25^ indicated the presence of oligomannose-type, hybrid-type and bi-antennary complex N-glycans in total material. PNGase F treatment, which specifically removes N-glycans, reduced ConA staining, confirming that human lung contains a variety of N-linked glycans (Fig. 1b). The presence of sialic acid was supported by staining with two commonly used sialic acid-recognizing lectins, *Sambucus nigra* agglutinin (SNA) and *Maackia amurensis* lectin-I (MAL-I)^26,27^. SNA, which detects the expression of α2,6-linked sialic acid, bound a wide range of proteins and the staining was not affected by neuraminidase S digestion, which hydrolyses α2,3-linked sialic acids (Fig. 1c). PNGase F partially reduced SNA binding, as α2,6-sialylation is also present on O-glycans. The expression of α2,3-sialylated glycans was assessed by the recognition with MAL-I. Treatment with PNGase F diminished staining by MAL-I, indicating that α2,3-sialylation predominantly occurs on N-glycans (Fig. 1d). Digestion with neuraminidase S significantly diminished staining, although neuraminidase A, which removes sialic acids in all linkages, did not result in complete elimination of MAL-I binding. This result with MAL-I is consistent with our recent finding that MAL-I can recognize some types of non-sialylated complex-type N-glycans^28^.

The expression of fucosylated glycans was detected by *Aleuria aurantia* lectin (AAL), which preferentially binds glycans containing α2-, α3-, α4- or α6-fucosylated sequences^29^. Fucosylation is predominantly present on N-glycans as PNGase F reduced almost all AAL binding (Fig. 1e). Because fucosylation is associated with the expression of the ABO and the Lewis blood groups, we further differentiated the expression of these glycan epitopes. Positive staining with *Ulex europaeus-*I (UEA-I), which binds the α2-fucosylated H-type 2 blood group^30^, showed exclusive expression of this modification on N-glycans (Fig. 1f). Staining with *Dolichos biflorus* agglutinin (DBA)^31^ indicated the absence of the blood group A antigen (Fig. 1g). The Lewis A (LeA) and sialyl-Lewis A (SLeA) antigens were exclusively detected on O-glycans, and not affected by PNGase F treatment, whereas the Lewis X (LeX) epitopes was mainly present on N-glycans, as PNGase F removed staining (Supplementary Fig. 1).

### Human lung expresses structurally diverse N-glycans

**Figure 2:**
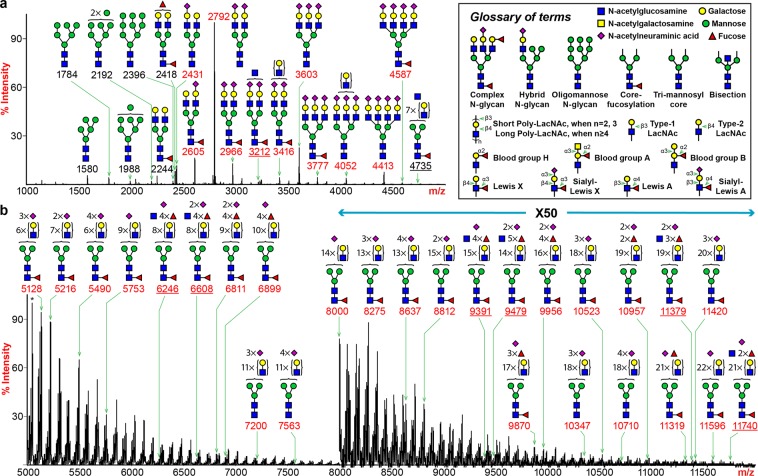
N-glycan MS profile of human lung. All molecular ions detected represent permethylated species and are present in the form of [M + Na]^+^. (**a**) MALDI-TOF-MS spectrum covers the m/z range between 1000 and 5000. (**b**) MALDI-TOF-MS spectrum covers the m/z range between 5000 and 12000. The relative intensity of the most abundant peak in each spectrum is set at 100%. The relative intensity of the highest peak in the panel (b), as indicated by an asterisk (*), equals 0.1% of the highest peak in panel (a), if both peaks are plotted in the same panel. Selected peak masses were annotated to reflect the diversity of structural features including sialylated glycans (red), non-sialylated (black) and bisected (underline) glycans. Cartoon symbols appear above a curly parenthesis indicates that the sequences corresponding to these compositions cannot be unequivocally defined with linkage information. Symbol representations of monosaccharides are: yellow circle, Galactose; green circle, Mannose; red triangle, Fucose; blue square, N-acetylglucosamine; purple diamond, N-acetylneuraminic acid.

To elucidate in-depth structural details of the human lung glycome, we performed mass spectrometric characterization on major glycan classes. The MALDI-TOF MS spectrum of human lung N-linked glycans released by PNGase F revealed striking structural variations up to m/z 12000 with more than 500 assignable peaks detected (Fig. 2; Supplementary Table 1). The overall profile was dominated by the peak at m/z 2792, which represented a bi-antennary, di-sialylated structure. A full set of oligomannose structures (m/z 1580, 1784, 1988, 2192 and 2396) was detected in the low mass region. Molecular ions consistent with a series of bi-, tri- and tetra-antennary complex-type glycans were observed to carry variable lengths of N-acetyllactosamine (LacNAc) repeats. The highest number of LacNAc units observed from our analysis was (LacNAc)_22_ (e.g., m/z 11596). Elongation of LacNAc units occurred exclusively in the linear form as we did not detect the presence of 3,6-subtituted Gal by GC-MS linkage analysis, which corresponds to branched PL chains (Supplementary Table 2). Mono-, di-, tri- and tetra-sialylation were observed and predominantly occurred in the form of Neu5Ac-LacNAc on the non-reducing ends. A minor form of sialylation was also detected where the sialic acid was linked to the GlcNAc (Supplementary Fig. 2). A significant proportion of complex type N-glycans was mono-fucosylated (e.g., m/z 2605, 2966, 3416 and 3777), a structural feature proposed to be core fucosylation with an α6-linkage and was confirmed with GC-MS linkage analysis (Supplementary Table 2**;** Fig. 2). In the higher m/z regions, the compositions of a series of complex glycans were consistent with multi-fucosylated structures with sialylation (e.g., m/z 6811, 6899, 9870 and 9956). Detection of those peaks indicated the expression of blood group H, LeA/X and SLeA/X antigens and was confirmed by tandem MS and GC-MS analyses (Supplementary Table 2**;** Fig. 2b,c). Molecular ions corresponding to structures with an additional HexNAc were observed and confirmed to be a bisecting GlcNAc by GC-MS experiment (Supplementary Table 2**;** Fig. 2a, e.g., m/z 3212, 4735, 6246, 6608, 9391 and 9479).

By comparing the relative intensities of peaks from the MALDI-TOF spectrum, we could dissect the patterns of human lung N-glycosylation in a semi-quantitative manner (Supplementary Fig. 2d). Sialylation represents a major modification as ~75% of the N-glycans carried at least one sialic acid. Of the sialylated species, ~60% of glycans were modified with two Neu5Ac residues. The proportions for tri- and tetra-sialylated structures were relatively minor. They together constituted about 10% of the total sialylated glycans. Although ~500 fucosylated species were identified from our analysis, in terms of relative quantities, only ~30% of the glycans were fucosylated. Approximately 80% of the fucosylated glycans displayed a single fucose. The proportion of glycans with a second fucose dropped sharply to 15% while the occurrences of tri-, tetra- and penta-fucosylation were dramatically lower. Nonetheless, a few structures with six (e.g., m/z 4823, 5185, 5273, 5722, 6083 and 6171) or seven (e.g., m/z 5447) fucose residues were detected with extremely low abundances. Lastly, we looked at the proportions of bisected glycans. Approximately 5% of the human lung N-glycans carried a bisecting GlcNAc. In contrast to the non-bisected glycans, sialylation was not a prominent terminal modification as only 25% of bisected glycans were sialylated. Conversely, most of bisected structures were fucosylated (80%).

### Human lung expresses both α2,3- and α2,6-sialylated N-glycans

**Figure 3:**
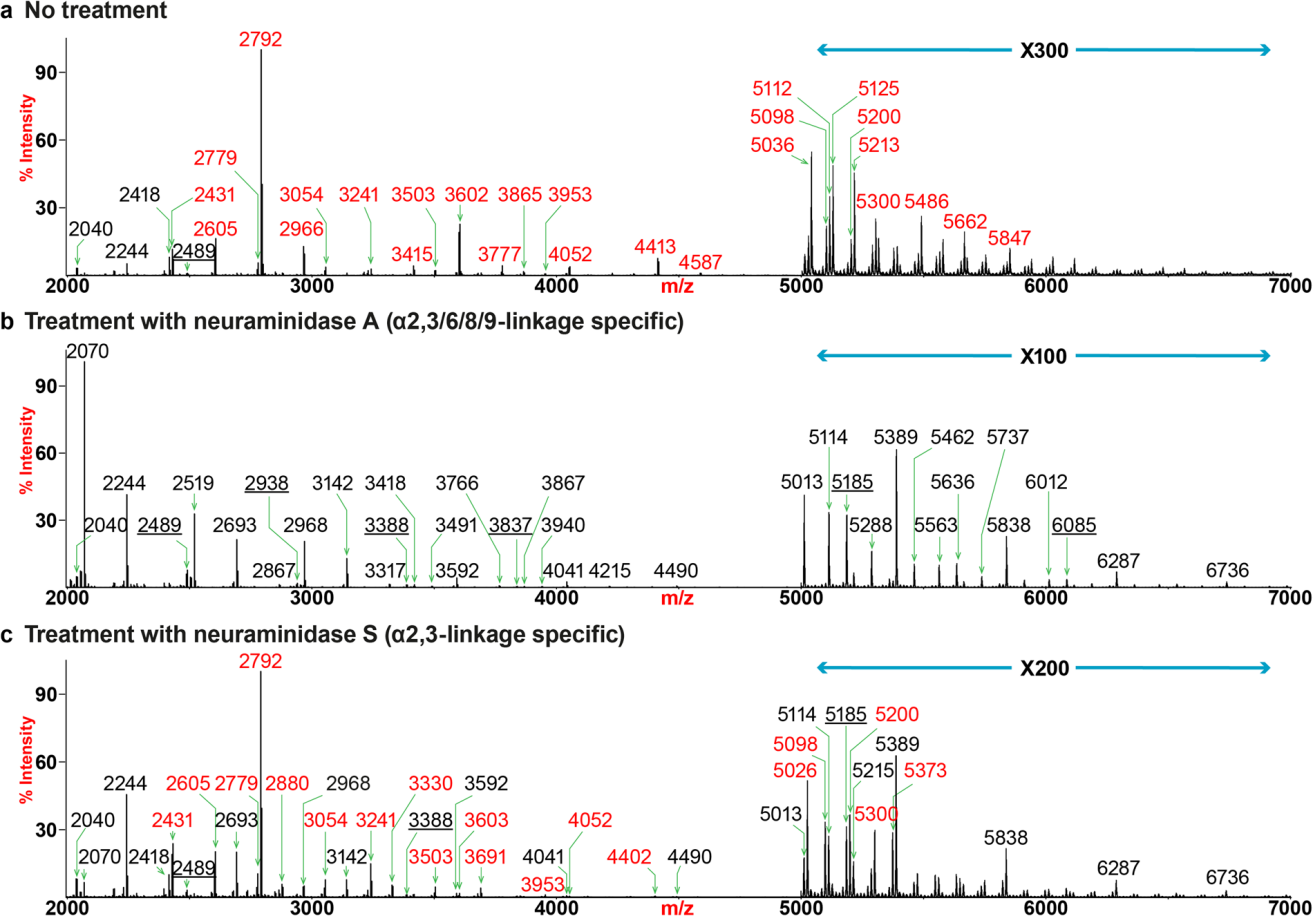
N-glycan MS profiles of human lung following neuraminidase treatment. All molecular ions detected represent permethylated species and are present in the form of [M + Na]^+^. (**a**) MALDI-TOF-MS spectrum of untreated human lung N-glycans. (**b**) MALDI-TOF-MS spectrum of human lung N-glycans treated with neuraminidase A. (**c**) MALDI-TOF-MS spectrum of human lung N-glycans treated with neuraminidase S. Peaks representing sialylated glycans are colored in red, non-sialylated glycans are in black and bisected glycans are underlined.

We further explored the linkage of sialic acids by treatment with neuraminidases A and S (Fig. 3**;** Supplementary Fig. 3). Digestion of human lung N-glycans with neuraminidase A resulted in complete de-sialylation (Fig. 3b**;** Supplementary Fig. 3b). The highest peak with the m/z at 2070 represented a bi-antennary structure without core fucosylation. The dramatic increase of this reaction product was consistent with the dominant expressions of its corresponding sialylated structures prior to the digestion (Fig. 3a, m/z 2431 and 2792). The compositions of the six most abundant molecular ions were consistent with fucosylated or non-fucosylated tri-mannosyl core structures carrying (LacNAc)_2–4_ repeats (m/z 2070, 2244, 2519, 2693, 2968 and 3142). As backbones for the addition of sialic acids, they constituted ~80% of the human lung N-glycome. Further extensions with LacNAc units led to the expression of glycans displaying PL motifs where the highest detectable number of repeats was (LacNAc)_23_ (Supplementary Fig. 3b, m/z 11684). A second series of backbone structures for sialylation included glycans carrying a bisecting GlcNAc (Fig. 3b, m/z 2489, 2938, 3388 and 3837). Although bisected glycans were estimated to make up as low as 5% of the total N-glycome, they can be elongated equally well with the highest detectable number of LacNAc repeat being (LacNAc)_22_ (Supplementary Fig. 3b, m/z 11481).

Compared to the profile of untreated lung, the bi-antennary, di-sialylated structure at m/z 2792 remained the most abundant glycan after the enzymatic treatment, indicating both sialic acids were mostly α2,6-linked (Fig. 3a,c). However, a small proportion of this structure also possesses α2,3-sialylation, as an increase in the relative intensity of its partially de-sialylated product at m/z 2431 was detected after the reaction. Other noticeable changes in the lower end of the spectrum included the emergence of the de-sialylated structures (e.g., m/z 2244, 2693, 3142 and 3241) due to partial or complete removal of sialic acids from their sialylated counterparts prior to the neuraminidase S digest (Fig. 3a; e.g., m/z 2966, 3602 and 3777). In the higher mass regions, the MALDI-TOF spectrum after neuraminidase S was more comparable to that of the neuraminidase A treated glycome than the control (Fig. 3a–c; m/z 5000–7000). This was exemplified by the appearance of prominent non-sialylated peaks, all of which were detected after the enzymatic digest by either of the enzyme (e.g., m/z 5013, 5114, 5185, 5389, 5838, 6287 and 6736). All the major sialylated species after neuraminidase S treatment corresponded to mono-sialylated glycans with α2,6-linkages (e.g., m/z 5026, 5098, 5200, 5300 and 5373). Patterns in the high mass regions demonstrated additional degrees of similarities between the profiles of glycans following neuraminidase A or S treatment (Supplementary Fig. 3a–c; m/z > 7000). The compositions of the major identified peaks were consistent with fully desialylated glycans carrying PL extensions (e.g., m/z 7190, 8089, 8988, 9887, 10786 and 11684). They were all detected with similar relative intensities after either neuraminidase A or S digestion. These results demonstrated that large extended N-glycans are mainly α2,3-sialylated. Some minor sialylated species with α2,6-sialylation were detectable and they all represented mono-sialylated structures with various degrees of fucosylation (e.g., m/z 7102, 7276, 7624, 7725, 8001, 8175 and 8450).

Since the short glycans were significantly more abundant than elongated glycans, we concluded that the human lung N-glycome contained a higher overall proportion of α2,6-sialylation, and these are mainly on shorter complex-type N-glycans, compared to α2,3-sialylated structures that are enriched on larger PL-containing complex-type N-glycans. The observation was also confirmed by GC-MS linkage analysis as a higher relative abundance was detected for the 6-substituted Gal than the 3-subsitituted Gal (Supplementary Table 2).

### Phosphorylated glycans are minor components of the human lung N-glycome

Our recent studies indicated that IAV can bind to some phosphorylated oligomannose-type N-glycans from the human lung^24^. To characterize such glycans, we isolated the phosphorylated glycans from a human lung by treating lung glycopeptides with Endo H, which releases oligomannose- and hybrid-type N-glycans (Supplementary Fig. 4a). The compositions of the five prominent molecular ions were consistent with Man_**5–9**_GlcNAc (m/z 1335, 1539, 1743, 1947 and 2151). Other detectable peaks represented neutral hybrid (e.g., m/z 1580, 1784, 1825, 2029 and 2233) and sialylated hybrid glycans (e.g., m/z 2390, 2595 and 2840). One extremely minor phosphorylated structure was identified with the composition of Man_6_GlcNAc-phosphate (m/z 1641). We further enriched the phosphorylated species by desialylating the Endo H released N-glycans and separating via anion exchange chromatography (Supplementary Fig. 4b). We detected a series of phosphorylated oligomannose-type glycans (m/z 1641, 1845, 1947, 2049, 2151 and 2253) as well as phosphorylated hybrid-type structures (m/z 1886, 2090, 2192, 2395 and 2845). The expression of phosphorylated glycans was confirmed by MS/MS experiments (Supplementary Fig. 4c,d), as the fragment ion at m/z 156 specifically correspond to a partially permethylated phosphate group ([NaPO^4^-CH_3_ + Na]^+^) rather than a potential sulfate group ([NaSO^4^ + Na]^+^).

### The O-glycome of human lung mainly expresses two sialylated structures

**Figure 4:**
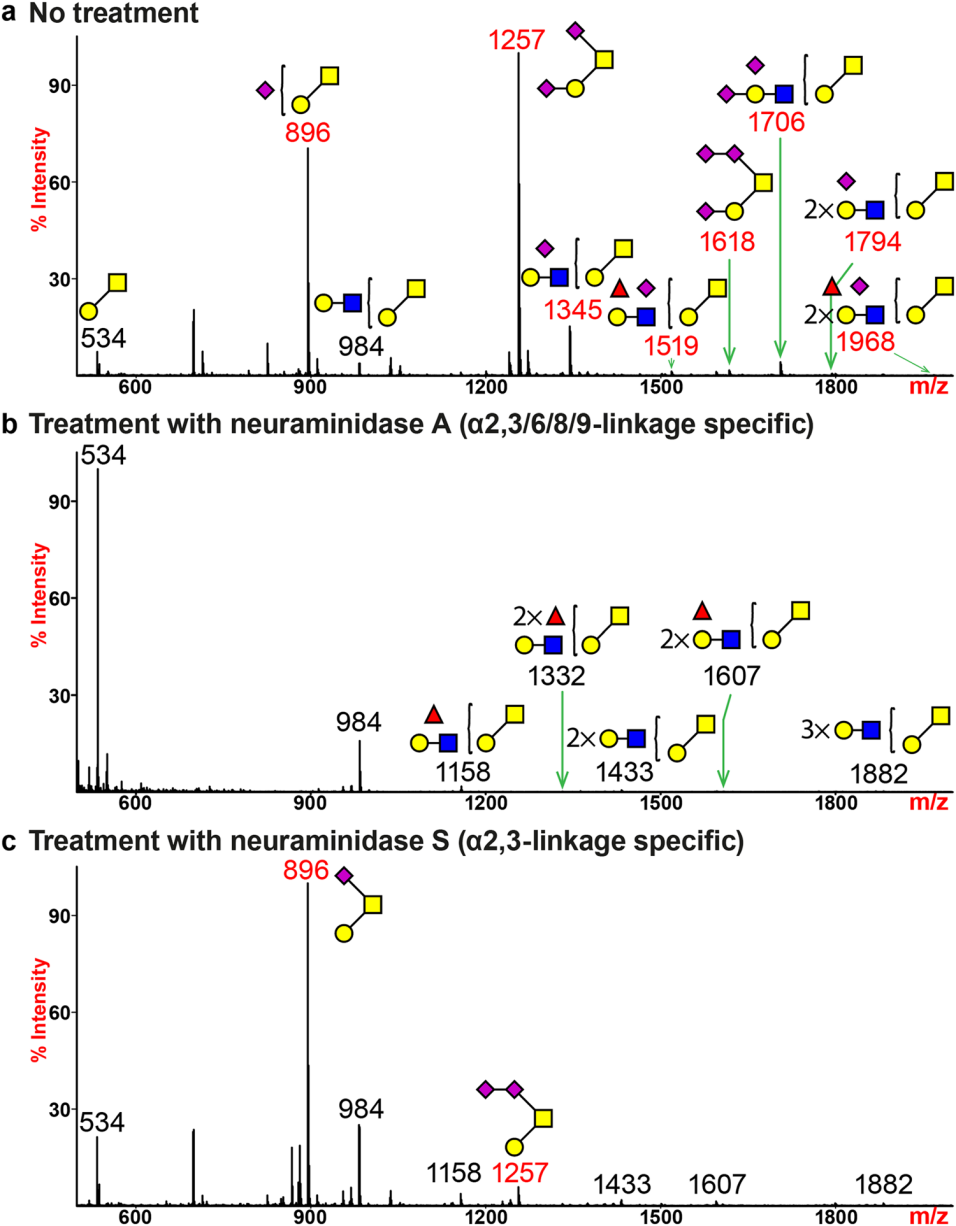
O-glycan MS profiles of human lung following neuraminidase treatment. All molecular ions detected represent permethylated species and are present in the form of [M + Na]^+^. (**a**) MALDI-TOF-MS spectrum of untreated human lung O-glycans. (**b**) MALDI-TOF-MS spectrum of human lung O-glycans treated with neuraminidase A. (**c**) MALDI-TOF-MS spectrum of human lung O-glycans treated with neuraminidase S. Peaks representing sialylated glycans are colored in red and non-sialylated glycans are in black. Symbol representations of monosaccharides are: yellow circle, Galactose; green circle, Mannose; red triangle, Fucose; blue square, N-acetylglucosamine; yellow square, N-acetylgalactosamine; purple diamond, N-acetylneuraminic acid.

The soluble mucins of the lung were removed by perfusion originally, and unavailable for analyses here, but we have previously analyzed mucins from normal donors and those with cystic fibrosis^32^. The major residual O-glycome was dominated by the expression of mono- and di-sialylated core-1 structures (Fig. 4a, m/z 896 and 1257; Supplementary Table 3). Molecular ions corresponding to structures with further addition of LacNAc units on the core disaccharide were observed to display sialic acids (m/z 1345, 1706 and 1794). Two fucosylated species (m/z 1519 and 1968) were detected, which were likely to carry the LeA and SLeA antigens, as indicated by the Western blot results and confirmed with MS/MS analysis (Supplementary Figs. 1 and 5a). A minor molecular ion which represented a tri-sialylated structure was detected (Fig. 4a, m/z 1618). MS/MS analysis indicated that the third sialic acid was added to the Neu5Ac on the 6-arm of the core GalNAc, forming a di-sialyl motif via a potential Neu5Acα2,8-Neu5Ac linkage (Supplementary Fig. 5b).

Neuraminidase A treatment fully removed sialic acids from O-linked glycans (Fig. 4b). A significant elevation in the relative intensity of the core di-saccharide (m/z 534) was due to complete de-sialylation of the most abundant mono- and di-sialylated structures (m/z 896 and 1257). One minor structure with two fucoses (m/z 1332) was detected after de-sialylation. A heterogeneous expression of α2,3-and α2,6-linkages of sialic acids was revealed by neuraminidase S treatment (Fig. 4c). A significant reduction in the relative intensity of the di-sialylated core structure (m/z 1257) indicated the sialic acid was α2,3-linked to the galactose prior to digestion. The core GalNAc was sialylated via an α2,6-linkage which could not be removed by neuraminidase S and therefore generated the mono-sialylated core structure (m/z 896) after the enzymatic digest. The residual peak that represented a di-sialylated structure at m/z 1257 confirmed the presence of a di-sialyl motif that was resistant to neuraminidase S treatment (Fig. 4c, m/z 1256).

### Glycosphingolipid-derived glycome expresses sialylated structures with linear elongation

**Figure 5:**
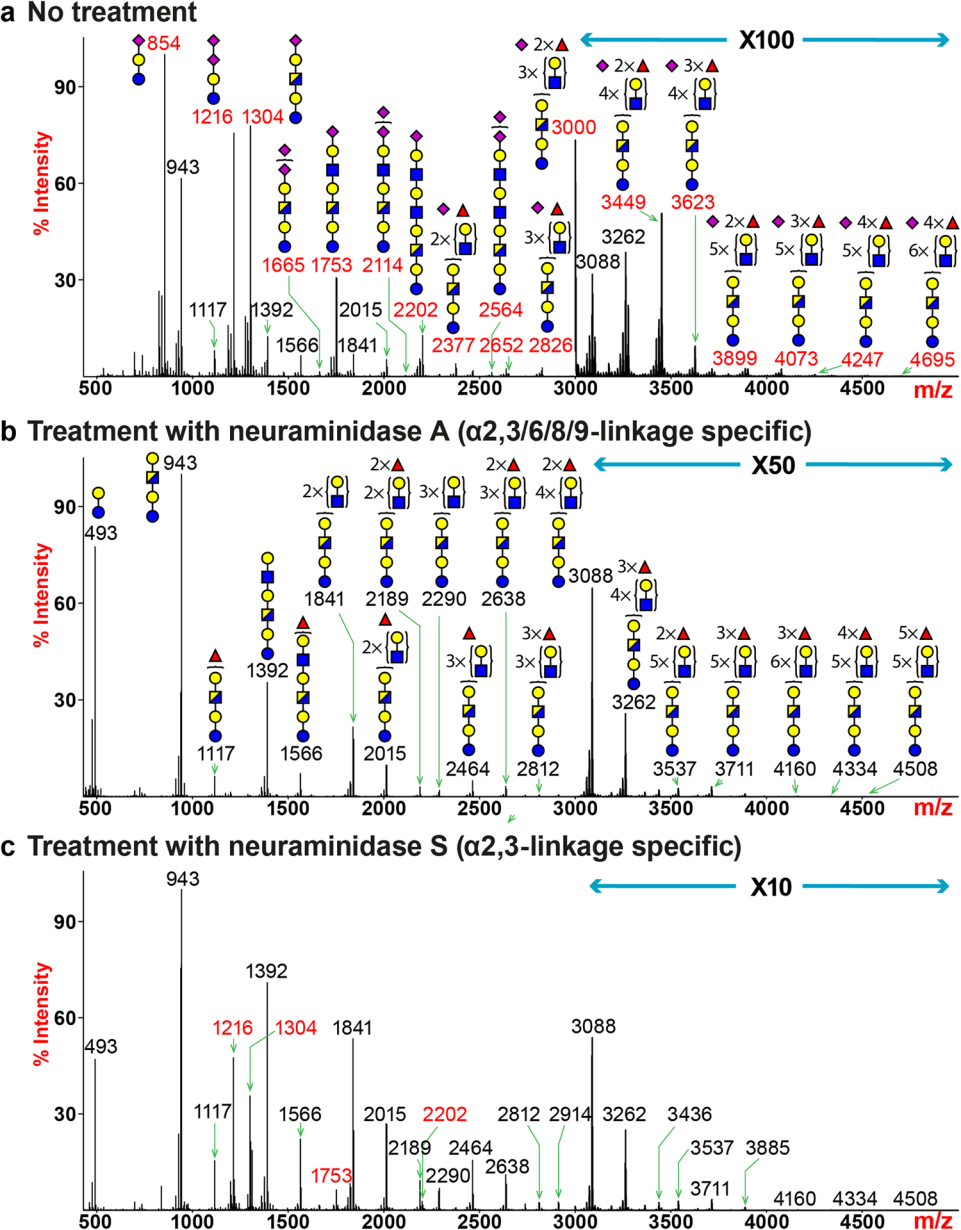
Glycosphingolipid (GSL)-glycan MS profiles of human lung following neuraminidase treatment. All molecular ions detected represent reduced, permethylated species and are present in the form of [M + Na]^+^. (**a**) MALDI-TOF-MS spectrum of untreated human lung GSL-glycans. (**b**) MALDI-TOF-MS spectrum of human lung GSL-glycans treated with neuraminidase S. (**c**) MALDI-TOF-MS spectrum of human lung GSL-glycans treated with neuraminidase A. Peaks representing sialylated glycans are colored in red and non-sialylated glycans are in black. Symbol representations of monosaccharides are: yellow circle, Galactose; red triangle, Fucose; blue square, N-acetylglucosamine; half-blue/half-yellow square, N-acetylhexosamine; purple diamond, N-acetylneuraminic acid.

Glycosphingolipids (GSLs) represent a major class of glycans that are present on the plasma membrane. Through MALDI-TOF MS analysis of human lung GSL-glycome, we detected more than 40 unique assignable peaks (Fig. 5a**;** Supplementary Table 4). Molecular ions consistent with the compositions of GM3 (ganglioside series, mono-sialylated glycan-3, m/z 854), GD3 (ganglioside series, di-sialylated glycan-3, m/z 1216) and GM1 (ganglioside series, mono-sialylated glycan-1, m/z 1304) represented the most abundant structures. The peak at m/z 943 corresponded to a structure with the composition of Gal-HexNAc-Gal-Glc, which represented the most abundant non-sialylated glycan (Fig. 5a). Sequential extensions of this tetra-saccharide core with variable lengths of PL repeats generated a series of unmodified (m/z 1392 and 1841), fucosylated (m/z 1117, 1566, 2015, 3088 and 3262) or sialylated (m/z 1753, 2202 and 2564) glycans. Towards the higher m/z region, molecular ions corresponding to sialylated structures with the addition of one or more fucoses were detected (m/z 2377, 2826, 3000, 3449, 3623, 3899, 4073, 4247 and 4695). MS/MS analysis of the molecular ion at m/z 3449 revealed that the LacNAc extensions beyond the Gal-HexNAc-Gal-Glc core occurred in a linear fashion, rather than branched (Supplementary Fig. 6a,b). Sialic acids only appeared on the non-reducing terminus in the form of Neu5Ac-LacNAc or SLeA/X antigen, with the latter isomeric structure being a minor population (Supplementary Fig. 6a). Additional fucose residues were linked to the repeating LacNAc units, generating an internal LeX motif, similar to what had been observed for human lung N-glycans. The common structural features of linear extension, terminal sialylation and the expression of internal LeX antigens were also shared by other glycans (e.g., m/z 2377, 2826 and 3000) and confirmed with MS/MS experiments.

Neuraminidase A digest completely removed sialic acids, revealing the pattern of LacNAc extension and fucosylation (Fig. 5b). The MALDI-TOF/TOF MS/MS spectrum of the molecular ion at m/z 3262 showed linear elongation of LacNAc units, as we observed fragment ions representing sequential addition of LacNAc or Fuc-LacNAc to the Gal-Glc core from the reducing end or to the terminal LeA/X antigen from the non-reducing end (Supplementary Fig. 6b). Neuraminidase S treatment fully desialylated GM3 (Fig. 5c, m/z 854), confirming the sialylation was in α2,3-linkage. In contrast, the outermost sialic acid of GD3 (m/z 1216) was in α2,8-linkage and therefore was resistant to hydrolysis. Partial desialylation of mono-sialylated glycans (m/z 1304, 1753 and 2202) indicated the expression of both α2,3- and α2,6-linkages. Glycans detected in the higher m/z region with four or more LacNAc units were completely desialylated, indicating those structures with PL extensions were entirely α2,3-sialylated prior to neuraminidase S digest (m/z 2377, 2564, 2826, 3000, 3449, 3623, 3899, 4073, 4247 and 4695). The pattern of differential sialylation recapitulated what had been observed for N-glycans where α2,6-sialylation preferentially occurs on smaller-sized glycans and larger-sized glycans with PL extensions are predominantly α2,3-sialylated.

### Characterization of a second human lung revealed similar patterns of glycosylation

**Figure 6:**
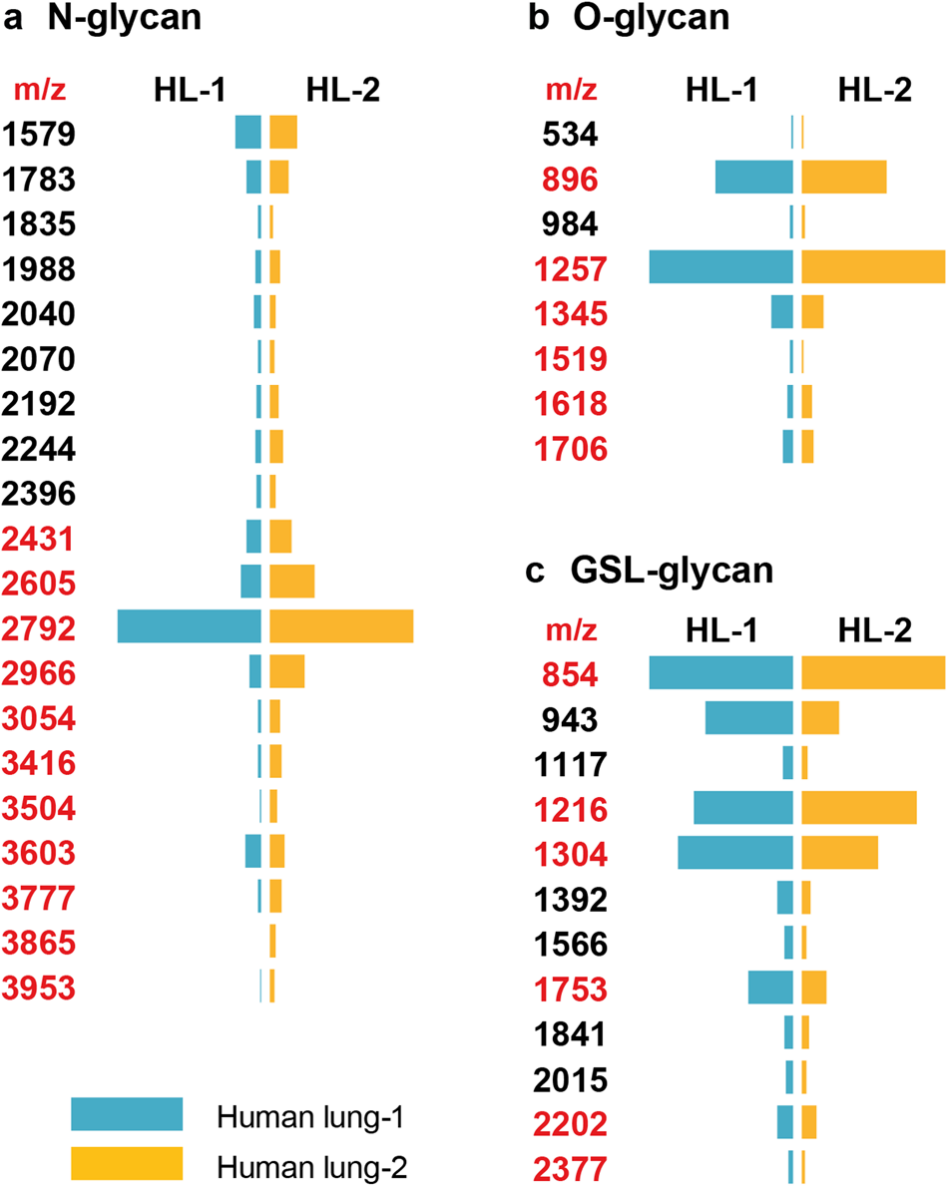
Comparison of glycosylation patterns between two human lungs. The relative intensities of selected N-glycans (**a**), O-glycans (**b**) and GSL-glycans (**c**) expressed by two human lungs are presented in a diverging bar chat. The length of a bar is proportional to the relative intensity (0–100%) of a peak detected on a MALDI-TOF-MS spectrum. A blue bar indicates the glycan is expressed by human lung-1 and an orange bar indicates the glycan is expressed by human lung-2. Peaks representing sialylated glycans are colored in red and non-sialylated glycans are in black.

To assess whether the glycosylation patterns we observed from one person represented common structural features in other individuals, we characterized the glycome of a second human lung. The Coomassie blue staining of the second lung showed similar patterns of protein expression to that of the first lung (Supplementary Fig. 7a). The expressions of sialylated and fucosylated structures were confirmed by staining with lectins and antibodies of homogenates from the second lung (Supplementary Fig. 7b–j). MS analysis revealed that the overall patterns of N-glycosylation were highly comparable between the two lungs with the bi-antennary, di-sialylated structure (m/z 2792) remained the most abundant glycan in the second lung (Fig. 6a, Supplementary Fig. 8a). Molecular ions consistent with a series of sialylated, fucosylated and bisected species were detected up to m/z 12000. As expected, some marginal differences were observed, which were likely due to person-to-person variations in the expression levels of glycosyltransferases (e.g., m/z 2605, 2966, 3054, 3416 and 3777). We did not detect unique composition that only belonged to one lung but not the other. The O-glycosylation patterns were almost identical between the two lungs (Fig. 6b, Supplementary Fig. 8b) and the profiles for GSL-glycans were also comparable (Fig. 6c, Supplementary Fig. 8c). Mono- and di-sialylated structures represented the major species (e.g., m/z 854, 1216, 1304, 1753 and 2202) while multi-fucosylation occurred on structures with variable PL repeats (e.g., m/z 3000, 3088, 3262, 3449, 3623 and 4073). The level of a non-sialylated glycan (m/z 943) was notably lower in the second lung. Overall, these results indicate that the glycosylation pattern of human lungs is likely to be similar between individuals, yet some degree of person-to-person variation could occur.

### Human lung tissue sections revealed staining with lectins and influenza viruses

**Figure 7:**
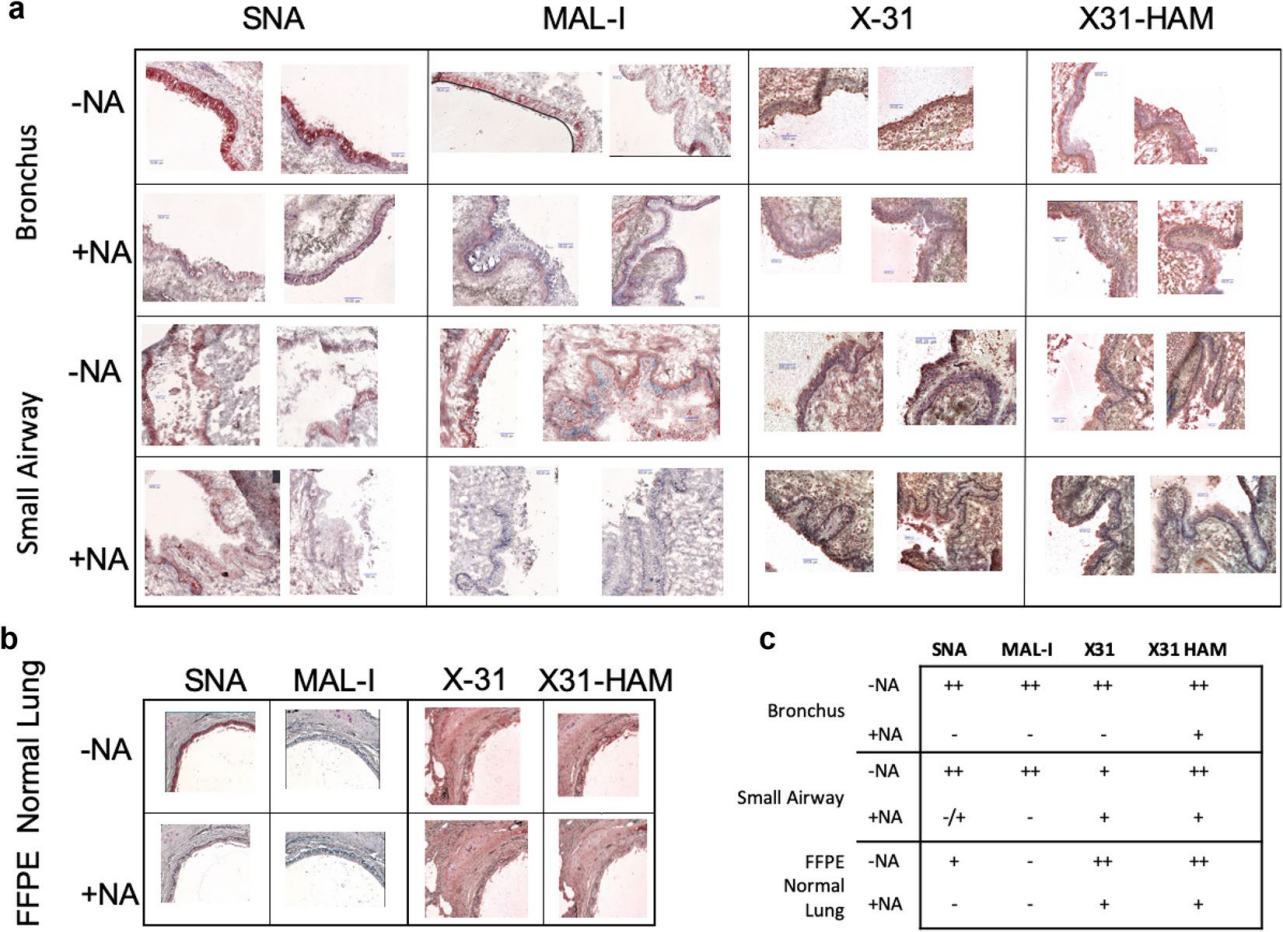
Immunohistochemistry staining of human lung sections. To visualize the localization of α2,3- and α2,6-linked sialic acids and the patterns of influenza virus attachment, human respiratory tissue sections were stained with lectins and influenza viruses. (**a**) Lectin-histochemistry staining images of frozen human bronchus and small airway sections with lectins SNA and MAL-I or with X-31 and X31-HAM strains of influenza viruses before (−NA) or after (+NA) neuraminidase treatment. (**b**) Lectin-histochemistry staining images of formalin fixed paraffin embedded (FFPE) human lung sections with lectins SNA and MAL-I or with X-31 and X31-HAM strains of influenza viruses before (−NA) or after (+NA) neuraminidase treatment. (**c**) Summary of immunohistochemistry staining results. ++, strong staining; +, moderate staining; −/+, weak staining; −, no staining.

The glycomics analysis examines the total lung glycome without specific localization to tissue types. Human lung tissue slides of the bronchus and small airways, from the donor organs prior to homogenization for glycomics analysis, were prepared for lectin histochemistry and pattern of virus attachment studies to aid in the characterization of sialylated receptors localized to these areas of the lung. On the frozen tissue slides (Fig. 7a), epithelial cells stained strongly without distinct localization for SNA for both tissue types, whereas MAL-I stained strongly on the basal surface with spotty staining at the apical edge in small airways. Bronchus epithelial cell staining is more uniform. Neuraminidase A (NA) treatment revealed that potential MAL-I binding to non-sialylated N-glycans does not provide a significant contribution to the overall lectin staining. Commercially available formalin fixed, paraffin embedded (FFPE) slides of normal human lung were used for comparison studies, and only SNA binding was detectable and was removed following NA treatment. MAL-I binding was not detected. Such discrepancies in results for frozen versus FFPE slides have previously been reported and could be due to the differences in slide preparation and fixation^23,33–39^ (Supplementary Table 5). It is possible that the frozen slides retain glycolipids, with a predominance of α2,3-linked Neu5Ac, that are removed in the fixation process for FFPE slides. It should also be noted that the *Maackia amurensis* preparation used in the various studies referenced here is not consistent; some studies use one variation of the lectin (MAL-I or MAL-II) and others utilize a combination (MAA).

Virus attachment experiments were performed to examine the surface attachment of IAV to the cells of the frozen and fixed lung tissue sections^40^ (Fig. 7b,c). We used X-31 and X-31 HAM viruses for these assays, as they exhibit strict sialic acid linkage preference for binding. X-31 recognizes α2,6- sialic acid and X-31 HAM recognizes α2,3- sialic acid^41^, thereby providing localization information in addition to broad confirmation of the presence of sialylated receptors. Both viruses also bind to all frozen and fixed tissue sections. Neuraminidase treatment effectively prevents staining by lectins to the frozen tissue slides, however some viral attachment is detectable following the same neuraminidase treatment conditions, revealing the possibility that the viruses are recognizing non-sialylated receptors in addition to the canonical sialylated ones which were removed by neuraminidase treatment^24^.

Antibodies against LeA, LeX, SLeA and SLeX were used in immunofluorescence experiments with frozen small airway sections (Supplementary Fig. 9). The anti-LeA antibody showed no staining to the epithelium of the small airway, while punctate staining was observed for anti-LeX and anti-SLeA antibodies. The antibody against SLeX exhibited strong staining to the epithelial cells corroborating MS data with the detection of SLeX epitope. Interestingly, while the LeA antibody recognized distinct glycoproteins on the Western blots, we did not detect staining to the epithelial cells via immunofluorescence, suggesting that such O-glycans are not predominant on such cells.

## Discussion

Here we present a comprehensive analysis of the natural N-, O- and GSL-glycans expressed by a human lung. Our MS data suggested that there appears to be a differential expression of α2,3- versus α2,6-sialylated glycans. N- and GSL-glycans found in a human lung are linearly extended with variable lengths of PL repeats with α2,6-sialylation predominantly occurring on short PL sequences and α2,3-sialylation on long PL sequences, perhaps revealing substrate preference for the α2,3- and α2,6-sialyltransferases. According to previous studies, the kinetic K_m_ value of the human α2,6-sialyltransferase-I (ST6Gal-I) towards a single type 2 LacNAc unit is significantly lower than that of the human α2,3-sialyltransferase-III, IV and VI (ST3Gal-III, IV and VI), which suggests that the ST6Gal-I may have a higher affinity towards short LacNAc sequences^42,43^. The crystal structure of ST6Gal-I further supports this speculation, as the active site of the enzyme has limited space to accommodate extended glycan sequences and the tri-mannosyl core of N-glycans appears to be critical for substrate recognition^44^. However, differential sialylation can also be affected by the expression levels of the sialyltransferases and their relative localizations within a cell.

The structural information of those natural glycans is crucial to understand the initial infection process by pulmonary pathogens that use glycans as host receptors. IAV strains can bind to sialic acid-terminating glycans expressed on the surface of human airway and the linkage difference of sialic acids is thought to be a key factor for the species barrier of influenza infection. This simple paradigm has evolved in recent years to include the glycan sequences beyond the terminal sialic acids. Sialylated branched N-glycans with elongated PL units have been proposed to be involved in IAV recognition^45–47^. Even though many studies have been using immuno-lectin histochemistry methods, glycan microarray platforms or computational tools to interrogate the molecular determinants governing the glycan-virus recognition, little structural information has been known for glycans naturally present in human respiratory tissues^21,23,45,48,49^. Therefore, our current understanding may not fully reflect the biological context of influenza infection on the surface of human airway. Our work confirmed the presence of α2,6-sialylated N-glycans with short LacNAc repeats (n ≤ 3) in human lungs. Those structures have been used in many glycan microarray studies and have shown binding to various strains of influenza viruses^45,50–53^. However, even though α2,6-sialylated N-glycans with extended PL (n > 3) sequences exhibit binding to H3N2 strains of influenza viruses^45^, there is weak evidence to support their expression by human, ferret or swine respiratory tissues^21,54,55^. On the contrary, α2,3-sialylated N-glycans with long PL (n ≥ 4) repeats, which were identified from the present work, are poor binders for influenza viruses^45,53^. Staining of human lung tissue slides indicates that neuraminidase treatment removing all sialic acid detectable by lectin binding does not prevent attachment of the influenza viruses X-31 and X-31 HAM. Moreover, our recent study identified a novel mode of glycan recognition by influenza viruses, which depends on phosphorylated glycans^24^. These results open up possibilities that influenza viruses may rely on multiple types of host receptors to adhere to respiratory epithelial cells and the infection process may be more complicated than currently supposed.

The tri-sialylated core 1 O-glycan has been associated with cytotoxicity of natural killer (NK) cells via inhibitory *cis-*interaction with Siglec-7 on cell surface^56,57^. The enzyme responsible for the addition of an α2,8-linked Neu5Ac on O-glycans has been shown to be human α2,8-sialyltransferase-VI and its expression level is enhanced in a human lung (www.proteinatlas.org). We speculate that a human lung may be armed with a huge number of tissue-resident NK cells to protect the lung from constant encounter of respiratory pathogens^58,59^. On the other hand, the enhanced expression of the tri-sialylated O-glycan may contribute to the hypo-responsiveness of NK cells so that they are not too damaging to lung tissues.

Our comprehensive characterization of human lung glycome also allows us to compare and verify animal models used in the study of influenza infection, from the angle of pulmonary glycosylation. By recruiting the same strategy of glycan release and MS analysis, the glycomic profiles of lungs from ferret, mouse and swine have been generated from previous studies using total lung organs as starting materials^54,55,60^. The ferret glycome has many structural features in common with the glycome of human lung, exhibiting a comparable MS profile, presence of moderate lengths of PL, exclusive expression of the Neu5Ac form^61^ and sialylation with a higher proportion towards α2,6- than α2,3-linked Neu5Ac. However, the expression of the Sda antigen (Neu5Acα2,3(GalNAcβ1-4)Galβ1,4GlcNAc) represents a major and unique structural feature of ferret lung glycome. The analysis of mouse lung revealed a highly comparable N-glycan profile to that of human lung, except the sialylation is predominantly in the form of Neu5Gc^60^. Despite the high degree of similarity, the mouse has been regarded as a poor animal model for the studies of influenza viruses, possibly due to poor binding affinity by influenza viruses against the Neu5Gc^62^. On the other hand, the glycome of a swine lung exhibited less similarity to a human lung, as the swine lung expresses high abundances of structures carrying the Neu5Gc and Galα1-3Gal non-human epitopes. However, swine are susceptible to infection by human, avian and swine influenza viruses. Additional factors such as the composition of pulmonary mucus may contribute to the difference in infectivity. Although not in the scope of the present study, human airway mucins are known to contain hundreds of sialylated, fucosylated and sulfated glycan structures^32^. In comparison to the data obtained from the present study, the O-glycome of a human lung is relatively simple.

In summary, the glycomics analysis of the human lung has generated a wealth of data with implications in influenza research, immunological studies, and technical advances including the analysis of phosphorylated glycans via MALDI-TOF MS. This work represents a beginning for the Human Glycome Project and paves the way for the study of other tissues and organs in the future.

## Materials and Methods

### Materials

Human donor lungs unfit for organ transplantation were provided by LifeLink Foundation, Inc. (Norcross, GA). The lungs were obtained through LifeLink, one of the approved non-profit organ procurements organizations, through informed and consented organ donation of deceased individuals and are not considered human subjects research. No personal identifying information was obtained on the donors, and falls under exemption category #4 (2016P000014), through the Beth Israel Deaconess Medical Center IRB. The lung#1 used in the present study for extensive glycomics analysis was obtained from a healthy 22-year old male without any pulmonary complications. The lung#2 was obtained from a healthy 19-year old male with minimal smoking history. Human lungs used for analyses were perfused before receipt and devoid of blood cells and soluble mucus. Materials were treated following ethical guidelines and regulations and approval for work with human tissues was obtained through Beth Israel Deaconess Medical Center and Harvard Medical School (COMS approval #15-251).

### Western blots

The human lung homogenate was prepared by blending the tissues in lysis buffer (25 mM TRIS, 150 mM NaCl, 5 mM EDTA, 1% CHAPS, pH 7.4), followed by sonication. Digestion of the homogenate by PNGase F, neuraminidase A and neuraminidase S was performed at 37 °C for 16 hours (New England Biolabs, Inc.). Proteins were stained with Coomassie Brilliant Blue (CBB) or transferred to a nitrocellulose membrane (Thermo Fisher Scientific). After blocking with 5% (w/v) bovine serum albumin (BSA) in TRIS-buffer saline with 0.05% TWEEN 20 (TBST) for 1 h at room temperature, Western and lectin blots were analyzed with anti-LeA (7LE, Santa Cruz Biotechnology), anti-SLeA (9L426, US Biological), anti-LeX (HI98, BioLegend, Inc.), biotinylated AAL, ConA, DBA, MAL-I, SNA, or UEA-I (Vector Laboratories) as a primary staining, and horseradish peroxidase (HRP)-labelled goat anti-mouse IgG antibody, goat anti-mouse IgM antibody, or streptavidin at 1:5000 dilution in TBST, using SuperSignal West Pico Chemiluminescent Substrate (Thermo Fisher Scientific).

### General procedures for glycan release

Detailed sample preparation procedures for glycomics analysis by mass spectrometry have been described previously^63^. In brief, frozen human lung tissue was homogenized and sonicated in ice-cold ultra-pure water. Glycolipids were extracted by the addition of methanol and chloroform whereas glycoprotein precipitants were collected by centrifugation. Glycans were released from GSLs via a recombinant endoglycoceramidase II (rEGCase II; Takara Bio USA Inc.) and reduced with sodium borohydride. Glycopeptides were reduced and carboxymethylated with dithiothreitol and iodoacetamide, followed by overnight trypsin digestion. N-linked glycans were released from glycopeptides by PNGase F and O-linked glycans by reductive elimination. All glycans were permethylated, chloroform extracted and purified by C18 Sep-Pak pre-packed columns (WAT054945, Waters Corp.) prior to mass spectrometric analysis.

### Neuraminidase digestion

To acquire the linkage information of sialylation, purified glycans were incubated with neuraminidase A (from *Arthrobacter ureafaciens*, P0722, New England Biolabs, Inc.) or neuraminidase S (from *Streptococcus pneumoniae*, P0743, New England Biolabs, Inc.) over a period of 24 hours at 37 °C.

### Sample preparation for phosphoglycomics

Following tryptic digestion of human lung homogenates, glycopeptides were treated with Endo H (from *Streptomyces picatus*, P0702, New England Biolabs, Inc.) over a period of 24 hours at 37 °C. Released glycans were separated from residual glycopeptides via C18 chromatography and were subsequently desialylated by mild acid hydrolysis (50 mM TFA, 20 min at 100 °C). Hydrolyzed glycans were fractionated into non-charged and charged pools via a strong-anion exchanger (QAE Sephadex A-25, GE Life Sciences). Neutral glycans were present in the flow-through and wash fractions (20 mM TRIS-base). Bound phosphorylated glycans were eluted with 100 mM NaCl in 20 mM TRIS buffer. Once dried, both charged and non-charged fractions can be permethylated directly.

### Data acquisition by mass spectrometer

The permethylated glycans were dissolved in 20 µl of methanol, from which 1 µl of the sample was mixed with 1 µl of the matrix (20 mg/ml of 2,5-dihydrobenzoic acid in 50% (v/v) aqueous methanol) and spotted onto a MTP 384 polished steel BC target plate (Bruker Daltonics). MALDI-TOF MS and MALDI-TOF/TOF MS/MS data were obtained from an ultrafleXreme mass spectrometer (Bruker Corp.) equipped with a Smartbeam II laser. Data acquisition was performed under positive mode via the software flexControl (version 3.4, build 135, Bruker Daltonics). Spectrum between mass-to-charge (m/z) 1000 and 8000 was acquired under reflectron mode whereas between m/z 5000 and 12000 was acquired under the linear mode. Monoisotopic masses are listed for compositions with m/z between 1000 and 7000 while average masses are listed for m/z between 5000 and 12000. The ProteoMass MALDI-MS calibration kit (MSCAL1, Sigma-Aldrich) was used to calibrate the MS mode. Each MS spectrum presented in this study represented an accumulated spectrum that was generated from 20,000 laser shots.

### Data processing and peak assignment

MS and MS/MS raw data was exported from flexAnalysis (version 3.4, build 76, Bruker Daltonics) as mass spectrum and was further processed by mMass^64^. Each mass spectrum was assigned and annotated manually with the aid of GlycoWorkBench^65^. The assignment of a glycan composition below m/z 7000 was based on the ^12^C isotopic composition of a selected peak as well as the knowledge of biosynthetic pathways of mammalian glycans. For peaks above m/z 7000, average mass was used to annotate the composition of a peak. Peak assignment was reassured by the detection of mass shift that corresponded to the composition of a LacNAc, a GlcNAc, a Fuc or a Neu5Ac. All N-glycans observed in this study were assumed to have a common core sequence of Manα1-6(Manα1-3)Manβ1-4GlcNAcβ1-4GlcNAc. All O-linked glycans were assumed to be mucin-type glycans with a reducing end GalNAc common core attached to Thr or Ser. All glycolipid-derived glycans were assumed to be the glycosphingolipid type glycans with a common core sequence of Galβ1-4Glc. Wherever possible, a proposed structure was further sequenced by MS/MS experiments to define additional structural information.

### Percentage calculation of structural features

The relative intensity of the most abundant peak in each spectrum was set at 100% and all other peaks were normalized accordingly. To generate the doughnut chart that illustrates the total percentage of a specific structural feature, the following equation was used:

$${\rm{Total}}\, \% \,{\rm{of}}\,{\rm{a}}\,{\rm{structural}}\,{\rm{feature}}=\frac{\sum \,{\rm{relative}}\, \% \,{\rm{of}}\,{\rm{all}}\,{\rm{molecular}}\,{\rm{species}}\,{\rm{that}}\,{\rm{contain}}\,{\rm{the}}\,{\rm{structural}}\,{\rm{feature}}}{\sum \,{\rm{relative}}\, \% \,{\rm{of}}\,{\rm{all}}\,{\rm{molecular}}\,{\rm{species}}}$$

The m/z values of molecular species used in the calculation range between 1000 and 7000.

### GC-MS linkage analysis

Detailed procedures have been previously described^63^. In brief, permethylated glycans were hydrolyzed in TFA before reduction by sodium borodeuteride and acetylation with acetic anhydride. The resulting partially permethylated alditol acetates were dissolved in hexane and analyzed by a Thermo Scientific TRACE 1310 Gas Chromatograph equipped with a Thermo Scientific Q Exactive Orbitrap mass spectrometry system. 2–3 µL of each sample was injected into an Agilent fused-silica capillary column of cross-linked DB-5MS (30 m × 0.25 mm × 0.25 µm). The GC conditions were as follows: inlet and transfer line temperatures, 290 °C; oven temperature program, 90 °C for 1 min, 8 °C/min to 290 °C for 5 min, 10 °C/min to 300 °C for 5 min; inlet helium carrier gas flow rate, 1 mL/min; split ratio, 10. The electron impact (EI)-MS conditions were as follows: ion source temperature, 300 °C; full scan m/z range, 30–750 Da; resolution, 60,000; AGC target, 1e6; maximum IT, 200 ms. Data were acquired and analyzed with Thermo TraceFinder 4.1 software package.

### Immuno-histochemistry staining

Frozen sections of human lung tissue were fixed in acetone: ethanol (75:25) prior to staining. Slides are pre-treated with hydrogen peroxide and blocked with 5% BSA in Phosphate-Buffered Saline with Tween-20 (PBST, 0.05% Tween). After 45 min washes in PBST, slides were incubated with biotinylated SNA or MAL-I (both at 20 ug/ml) overnight at 4 °C. The lectins were removed and the slides washed in PBST prior to incubation in Streptavidin-HRP (1:1000) for 1 hour at room temperature. A Tyramide Signal Amplification (TSA) kit was used to amplify the HRP signal^34^. Patterns of IAVs attachment were conducted in a similar manner with the blocking step using 5% normal goat serum in PBST and FITC-labelled virus^40^ as the primary. Rabbit anti-FITC HRP was used for secondary detection, TSA TMR reagent was applied for 10 min and the signal was developed for 2 min in AEC. Cells were counter stained with hematoxylin. Formalin fixed paraffin embedded slides (US Biomax, Inc.) were deparaffinized with xylene and rehydrated with an ethanol gradient. Antigen retrieval was completed by microwaving the slides in citrate buffer (0.1 M citrate acid, 0.1 M sodium citrate, pH 6.0) for 15 min. Slides were then stained in the same way as the frozen section slides.

### Immunofluorescence staining with antibodies

The double immunofluorescence antigen labelling of frozen human lung sections was performed with LeA, LeX, SLeA and SLeX, with EpCAM antibody. The frozen human lung sections were fixed in Acetone/Ethanol (75:25) for 10 min and washed twice with TBS. The sections were blocked with 5% normal donkey serum (Jackson ImmunoResearch Lab Inc, West Grove PA) for an hour at room temperature. The sections were incubated with rabbit anti-EpCAM (1:300, Abcam, Cat#: ab32392) plus (1) FITC conjugated mouse anti-LeA (1:100, Santa Cruz, cat#: sc-51512) and (2) FITC conjugated mouse anti-human CD15 (LeX, 1:50); (3) mouse anti-SLeA (1:500, CA19.9) and (4) Rat anti-SLeX (1:200, BD 555946) overnight at 4 °C. The slides were washed three times in TBS and incubated with Alexa 647 conjugated donkey anti-rabbit secondary antibodies (Jackson ImmunoResearch Lab, 1:300) for (1) and (2) and CY3 conjugated donkey anti-mouse (Jackson ImmunoResearch Lab, 1:300) and Alexa 647 conjugated donkey anti-rabbit secondary antibodies (Jackson ImmunoResearch Lab, 1:300) for (3) and CY3 conjugated donkey anti-rat (Jackson ImmunoResearch Lab, 1:300) and Alexa 647 conjugated donkey anti-rabbit secondary antibodies (Jackson ImmunoResearch Lab, 1:300) for (4). Samples were then washed three times with TBS and mounted with Prolong Gold anti-fade mounting media containing DAPI (Invitrogen).

### Reporting summary

Further information on research design is available in the Nature Research Reporting Summary linked to this article.

## Supplementary information

Supplementary Information.

## Data Availability

All data generated or analyzed during this study are included and in this published article and its Supplemental Information files.
