# Supplementary material for: The Human Lung Glycome Reveals Novel Glycan Ligands for Influenza A Virus
Source: Sci Rep. 2020 Mar 24;10:5320. doi: 10.1038/s41598-020-62074-z (PMC7093477; doi:10.1038/s41598-020-62074-z)
Supplement: Supplementary file 1 — Supplementary Information. [file 41598_2020_62074_MOESM1_ESM.pdf]

Supporting information for

The Human Lung Glycome Reveals Novel Glycan Ligands for Influenza A Virus

Nan Jia[1,†], Lauren Byrd-Leotis[1,3, †], Yasuyuki Matsumoto[1], Chao Gao[1,3], Alexander N. Wein[2], Jenna L. Lobby[2], Jacob E. Kohlmeier[2], David A. Steinhauer[2,3,*], Richard D. Cummings[1,3,*]

[1]Beth Israel Deaconess Medical Center, Department of Surgery and Harvard Medical School Center for Glycoscience, Harvard Medical School, Boston, MA, USA
[2]Department of Microbiology and Immunology, Emory University School of Medicine Atlanta, GA, USA
[3]Emory-UGA Center of Excellence of Influenza Research and Surveillance, (CEIRS) Atlanta GA, USA

*Corresponding Authors:
David Steinhauer: dsteinh@emory.edu
Richard Cummings: rcummin1@bidmc.harvard.edu

†Contributed equally.

Supplementary Figures S1-S9

Supplementary Tables S1-S5

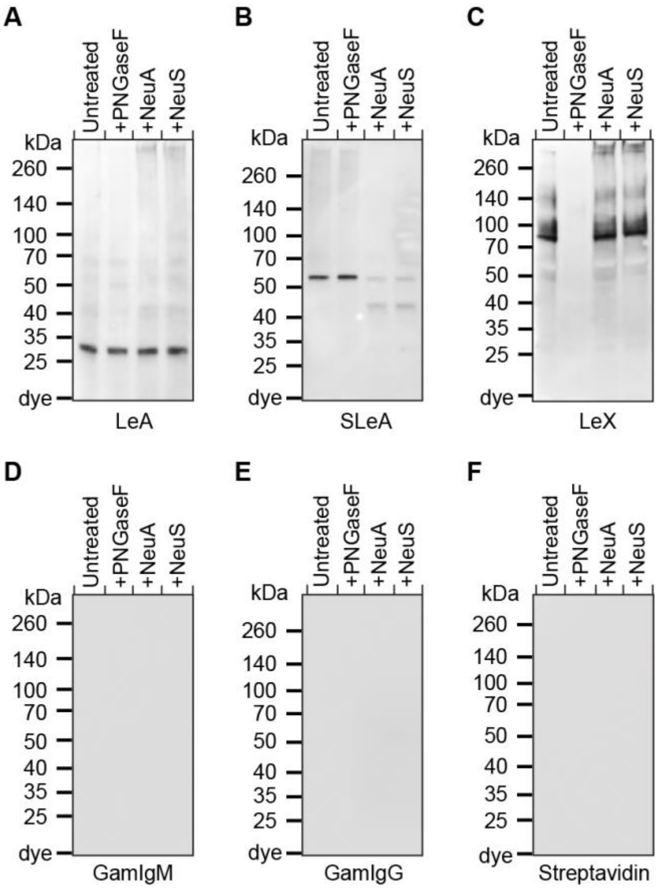

Supplementary Fig. 1 Western blot of human lung for detection of Lewis glycan epitopes. Tissue homogenate in human lung were treated with PNGase F (PNGaseF), Neuraminidase A (NeuA), or Neuraminidase S (NeuS), and separated by SDS-PAGE. The gel was analyzed by Western blot using antibodies against LeA (a), SLeA (b) and LeX (c). HRP-conjugated goat anti-mouse IgM (d), goat anti-mouse IgG (e), and Streptavidin (f) were used as quality control.

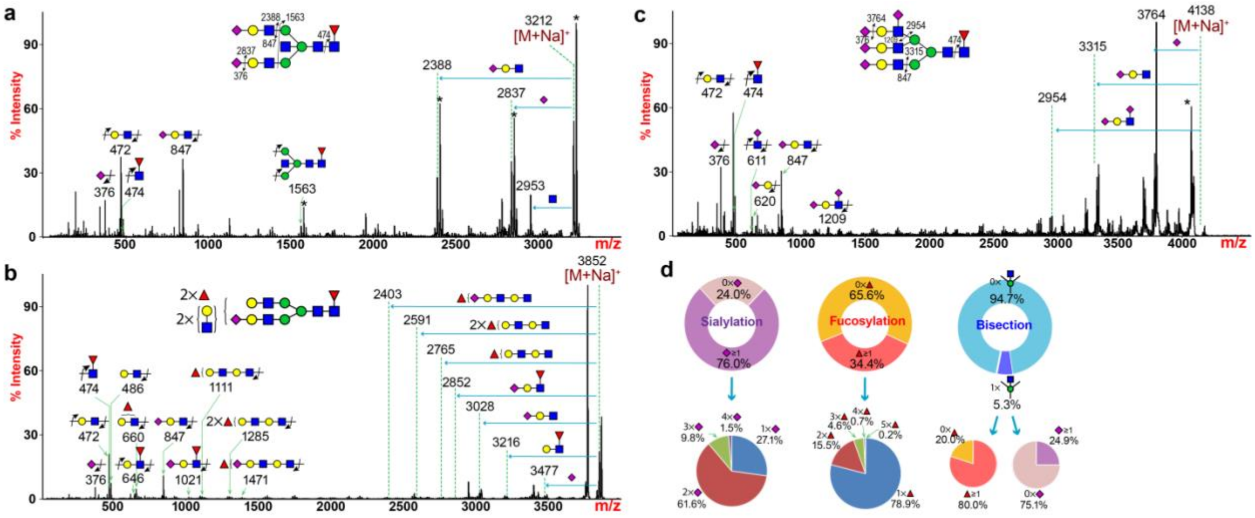

Supplementary Fig. 2 Further structural interrogation of human lung N-glycans. (a) MALDI-TOF/TOF-MS/MS analysis of a permethylated human lung N-glycan at m/z 3212 confirmed the expression of a bisecting GlcNAc. The fragment ion at m/z 1563 was generated from double-cleavage fragmentation, which indicated a GlcNAc residue was attached to the tri-mannosyl core. The loss of a terminal GlcNAc was confirmed by the detection of Y-ion at m/z 2953. (b) MALDI-TOF/TOF-MS/MS analysis of a permethylated human lung N-glycan at m/z 3852 confirmed the expression of Lewis antigens. The B-ion at m/z 660 represented a fucosylated LacNAc trisaccharide fragment ion, which indicated the expression of an H antigen or a terminal LeX antigen. The expression of SLeX was also detected which was confirmed by the B-ion at m/z 1021 together with its corresponding Y-ion at 2852. Detection of a double cleavage ion at m/z 646 suggested the presence of internal LeX motif as well as SLeX. (c) MALDI-TOF/TOF-MS/MS analysis of a permethylated human lung N-glycan at m/z 4138 confirmed the Neu5Ac-GlcNAc sequence. The addition of a fourth sialic acid on the GlcNAc of a LacNAc unit was confirmed by the presence of the fragment ion at m/z 611. The MS/MS spectra of all three molecular ions confirmed core-fucosylation as illustrated by the fragment ion at m/z 474. (d) The doughnut charts represented the relative quantitation of human lung complex N-glycans with sialylation, fucosylation or bisection. The percentage was defined as the sum of relative intensities carrying that specific structural feature divide by the sum of relative intensities of total glycans. Glycans with more than one sialic acid or fucose residue were further dissected to reflect the proportions carrying various numbers of sialic acid or fucose residues. Bisected glycans were further inspected to show the proportions of sialylated and fucosylated species.

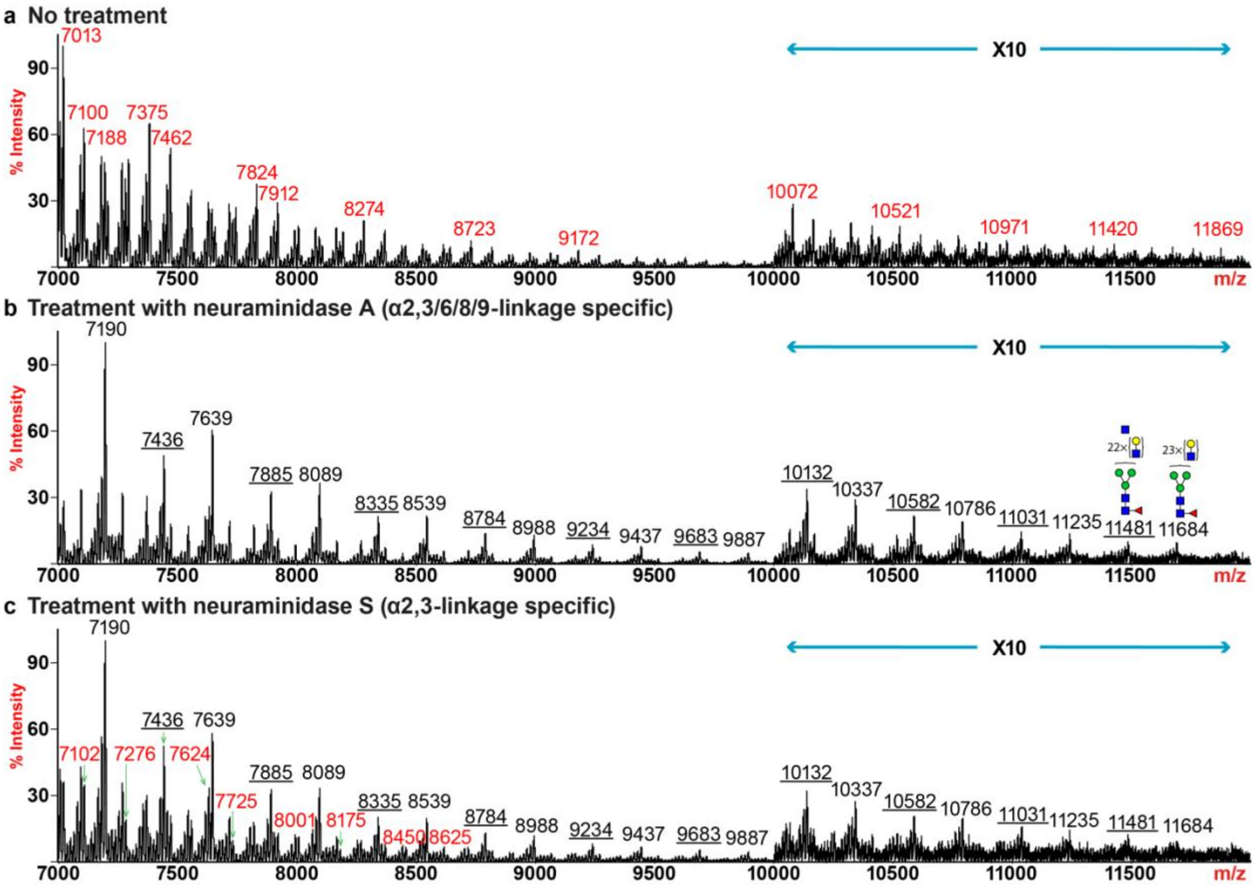

Supplementary Fig. 3 N-glycan MS profiles of human lung following neuraminidase treatment. The spectra cover the m/z region between 7000 and 12000. All molecular ions detected represent permethylated species and are present in the form of $[M+Na]^+$. (a) MALDI-TOF-MS spectrum of untreated human lung N-glycans. (b) MALDI-TOF-MS spectrum of human lung N-glycans treated with neuraminidase A. (c) MALDI-TOF-MS spectrum of human lung N-glycans treated with neuraminidase S. Peaks representing sialylated glycans are colored in red, non-sialylated glycans are in black and bisected glycans are underlined.

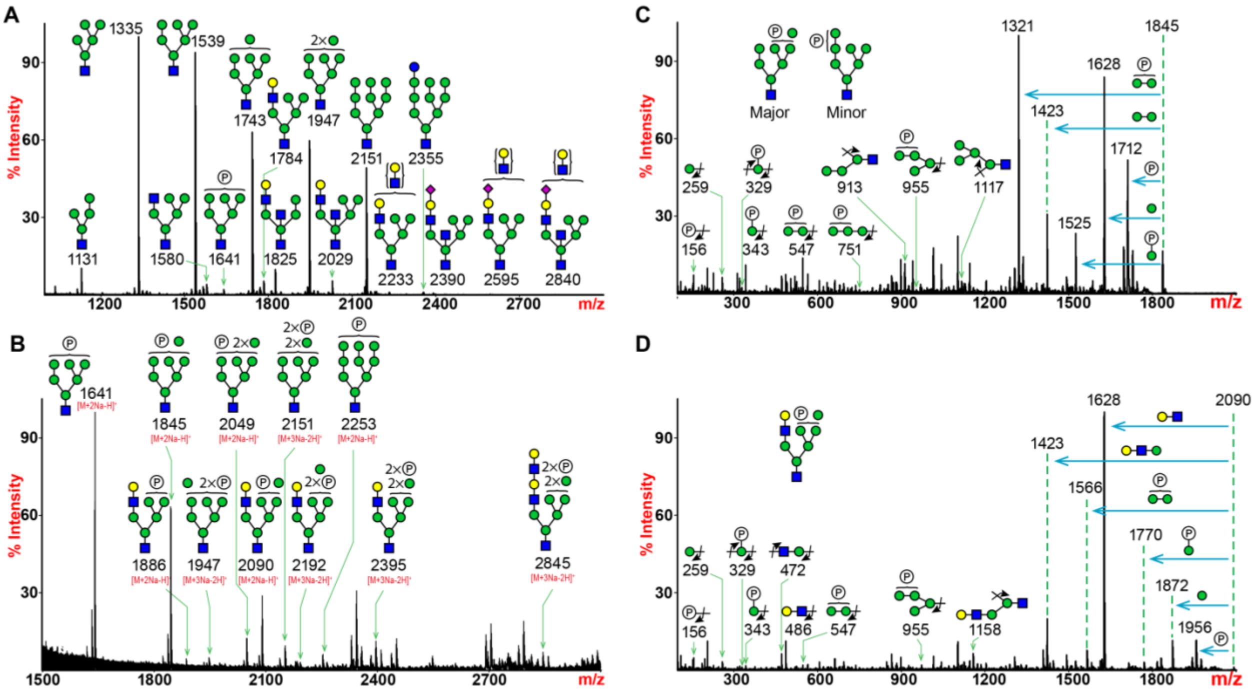

Supplementary Fig. 4 MS analysis of phosphorylated oligomannose glycans from a human lung. All molecular ions detected represent permethylated species and are present in the form of singly charged ions with sodium ion adducts. (a) MALDI-TOF-MS spectrum of human lung N-glycans released by an endoglycosidase Endo H, which releases oligomannose- and hybrid-type N-glycans. (b) MALDI-TOF-MS spectrum of human lung phosphorylated N-glycans released by Endo H, followed by de-sialylation and anion exchange chromatography. A phosphate group is symbolled by a circled letter ‘P’. (c) MALDI-TOF/TOF-MS/MS analysis of a permethylated human lung N-glycan at m/z 1845 confirmed the expression of a phosphorylated oligomannose-type structure. (d) MALDI-TOF/TOF-MS/MS analysis of a permethylated human lung N-glycan at m/z 2090 confirmed the expression of a phosphorylated hybrid-type structure.

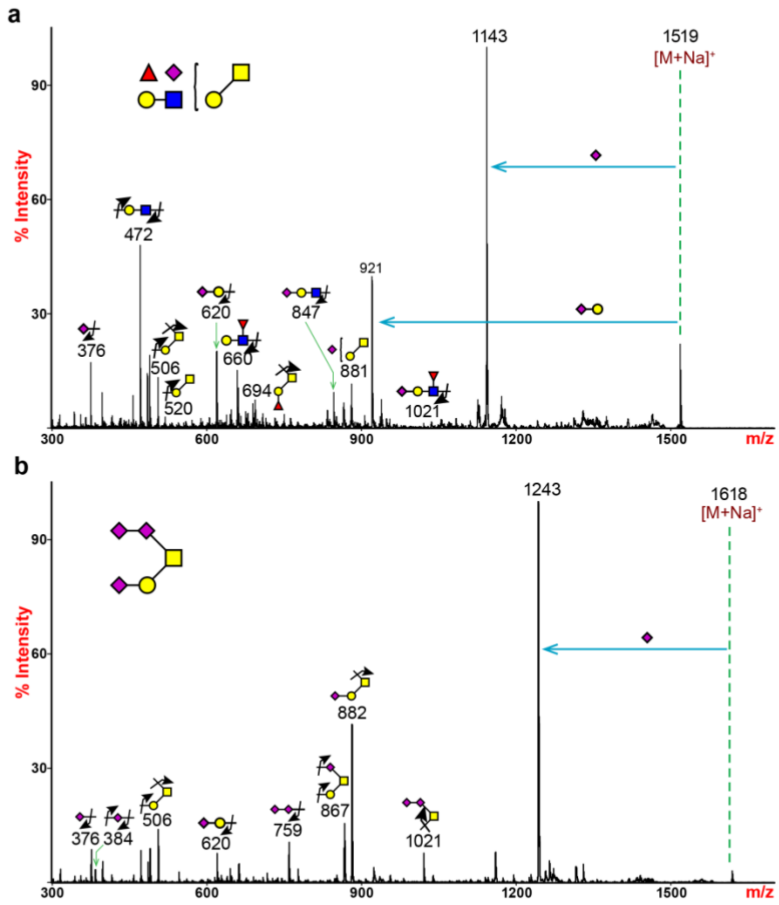

Supplementary Fig. 5 MALDI-TOF/TOF-MS/MS analysis of human lung O-glycans. (a) MALDI-TOF/TOF-MS/MS analysis of a permethylated human lung O-glycan at m/z 1519 confirmed the expression of Lewis antigens. (b) MALDI-TOF/TOF-MS/MS analysis of a permethylated human lung O-glycan at m/z 2090 confirmed the expression of a tri-sialylated core 1 structure.

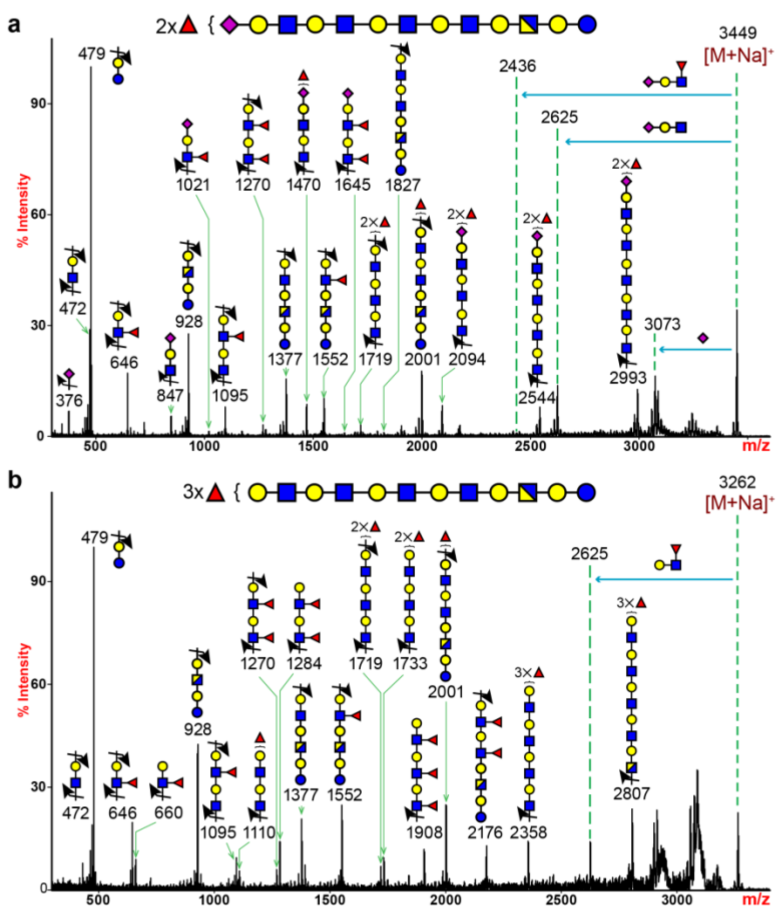

Supplementary Fig. 6 MALDI-TOF/TOF-MS/MS analysis of human lung GSL-glycans. (a) MALDI-TOF/TOF-MS/MS analysis of a permethylated human lung GSL-glycan at m/z 3449 confirmed the expression of Lewis antigens. (b) MALDI-TOF/TOF-MS/MS analysis of a permethylated human lung GSL-glycan (m/z 3262) after neuraminidase A treatment confirmed the expression of linearly extended GSL-glycans with Lewis antigens.

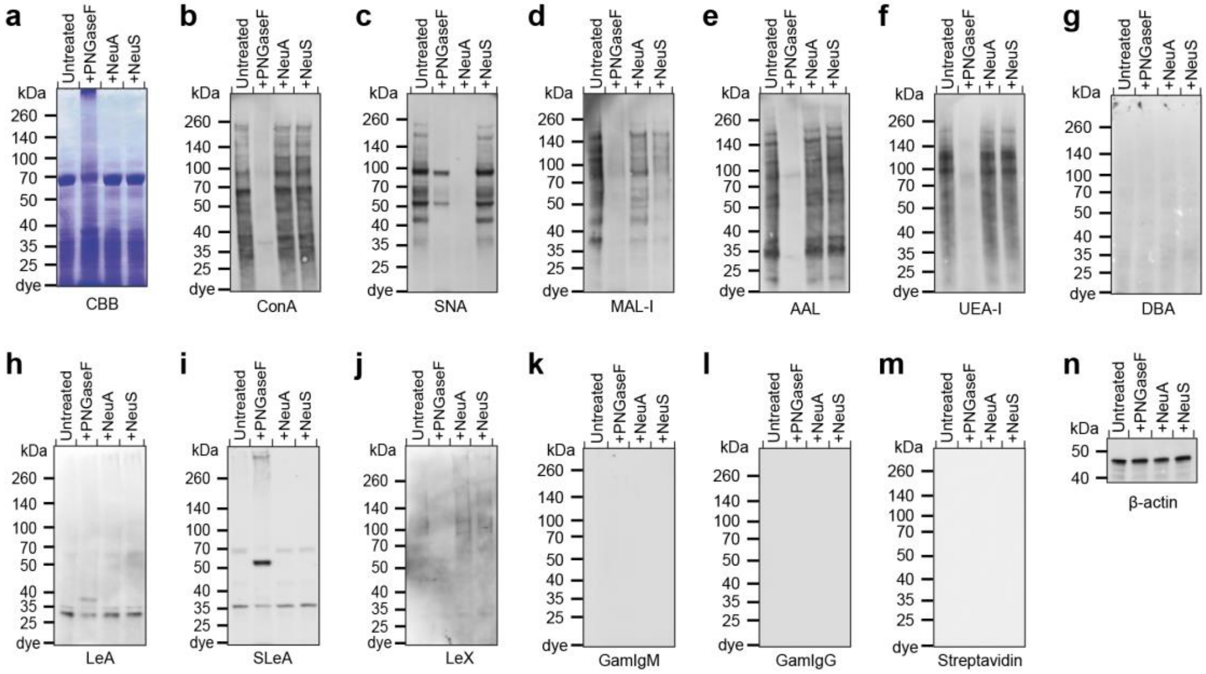

Supplementary Fig. 7 Western blot of human lung-2. Tissue homogenate obtained from human lung-2 were treated with PNGase F (PNGaseF), Neuraminidase A (NeuA), or Neuraminidase S (NeuS) and separated by SDS-PAGE. The gel was stained with Coomassie Brilliant Blue solution (a). Western blot was performed on the second human lung homogenate using lectins ConA (b), SNA (c), MAL-I (d), AAL (e), UEA-I (f), DBA (g) as well as antibodies against LeA (h), SLeA (i) and LeX (j). HRP-conjugated goat anti-mouse IgM (k), goat anti-mouse IgG (l), Streptavidin (m) and β-actin antibody (n) were used as quality control.

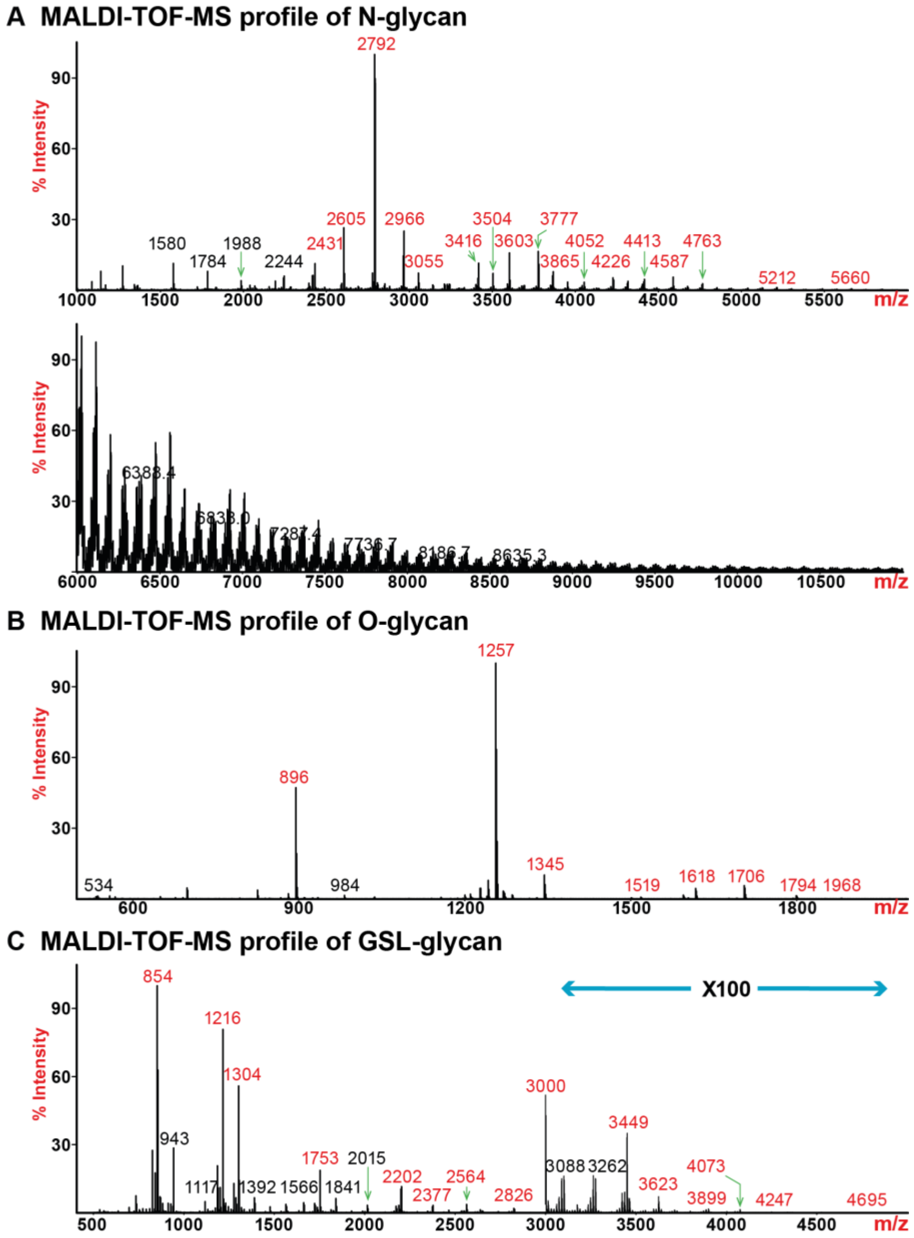

Supplementary Fig. 8 Comparison of glycosylation patterns between two human lungs. (a) MALDI-TOF-MS spectrum of untreated human lung-2 N-glycans. (b) MALDI-TOF-MS spectrum of human lung-2 O-glycans (c) MALDI-TOF-MS spectrum of human lung-2 GSL-glycans. Peak values representing sialylated glycans are colored in red and non-sialylated glycans are in black.

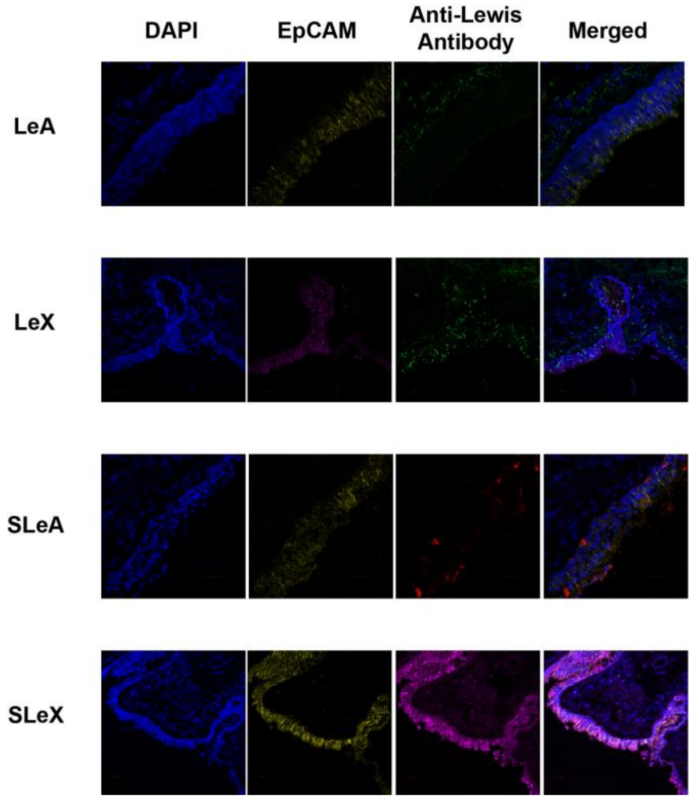

Supplementary Fig. 9 Immunofluorescence staining of human lung sections. The localization of Lewis antigens were visualized by immunofluorescence staining of human lung sections. Tissue sections were counterstained with a nucleus marker DAPI and an epithelium marker EpCAM.

Supplementary Table 1 List of assigned peaks of human lung N-glycans from MALDI-TOF-MS analysis

PNGase F released N-glycan (1000-7000)

Composition	Observed monoisotopic Mass (m/z)	Relative abundance (%)
Fuc_1 Hex_2 $HexNAc_2$	1141.4	3.51
Hex_3 $HexNAc_2$	1171.6	0.68
Fuc_1 Hex_3 $HexNAc_2$	1345.6	0.98
Hex_5 $HexNAc_2$	1579.8	8.08
Fuc_1 Hex_3 $HexNAc_3$	1590.8	0.24
Hex_4 $HexNAc_3$	1620.8	0.05
Hex_6 $HexNAc_2$	1783.9	6.21
Fuc_1 Hex_4 $HexNAc_3$	1794.9	0.17
Fuc_1 Hex_3 $HexNAc_4$	1836.0	1.68
Hex_4 $HexNAc_4$	1866.0	0.16
Hex_3 $HexNAc_5$	1907.0	0.10
Fuc_2 Hex_4 $HexNAc_3$	1968.0	0.19
$NeuAc_1$ Hex_4 $HexNAc_3$	1982.0	0.28
Hex_7 $HexNAc_2$	1988.0	3.11
Fuc_1 Hex_4 $HexNAc_4$	2040.1	4.77
Hex_5 $HexNAc_4$	2070.0	1.61
Fuc_1 Hex_3 $HexNAc_5$	2081.1	0.36
Hex_4 $HexNAc_5$	2111.1	0.21
$NeuAc_1$ Fuc_1 Hex_4 $HexNAc_3$	2156.1	0.55
$NeuAc_1$ Hex_5 $HexNAc_3$	2186.1	0.19
Hex_8 $HexNAc_2$	2192.1	2.60
Fuc_2 Hex_4 $HexNAc_4$	2213.1	0.25
$NeuAc_1$ Hex_4 $HexNAc_4$	2227.1	0.20
Fuc_1 Hex_5 $HexNAc_4$	2244.1	6.80
Hex_6 $HexNAc_4$	2274.1	0.22
Fuc_1 Hex_4 $HexNAc_5$	2285.1	0.85
Hex_5 $HexNAc_5$	2315.2	0.29
$NeuAc_1$ Hex_6 $HexNAc_3$	2390.2	0.55
Hex_9 $HexNAc_2$	2396.2	2.29
$NeuAc_1$ Fuc_1 Hex_4 $HexNAc_4$	2401.2	0.93
Fuc_2 Hex_5 $HexNAc_4$	2417.3	8.87
$NeuAc_1$ Hex_5 $HexNAc_4$	2431.2	13.66
Fuc_1 Hex_5 $HexNAc_5$	2489.3	1.11
Hex_6 $HexNAc_5$	2519.3	0.18
Fuc_3 Hex_5 $HexNAc_4$	2591.3	1.05
$NeuAc_1$ Fuc_1 Hex_5 $HexNAc_4$	2605.3	18.00
$NeuAc_1$ Hex_6 $HexNAc_4$	2635.3	0.39
$NeuAc_1$ Fuc_1 Hex_4 $HexNAc_5$	2646.3	0.39
Fuc_2 Hex_5 $HexNAc_5$	2663.3	0.30
$NeuAc_1$ Hex_5 $HexNAc_5$	2676.3	0.21

$Fuc_1\ Hex_6\ HexNAc_5$	2693.4	0.93
$Fuc_1\ Hex_5\ HexNAc_6$	2734.4	0.22
$NeuAc_1\ Fuc_2\ Hex_5\ HexNAc_4$	2779.4	7.81
$NeuAc_2\ Hex_5\ HexNAc_4$	2792.4	100.00
$Fuc_3\ Hex_5\ HexNAc_5$	2837.4	0.19
$NeuAc_1\ Fuc_1\ Hex_5\ HexNAc_5$	2850.4	1.41
$Fuc_2\ Hex_6\ HexNAc_5$	2867.5	0.14
$NeuAc_1\ Hex_6\ HexNAc_5$	2880.4	1.05
$Fuc_1\ Hex_6\ HexNAc_6$	2938.4	0.27
$NeuAc_1\ Fuc_3\ Hex_5\ HexNAc_4$	2953.5	0.21
$NeuAc_2\ Fuc_1\ Hex_5\ HexNAc_4$	2966.5	11.10
$NeuAc_1\ Fuc_2\ Hex_5\ HexNAc_5$	3024.5	0.07
$NeuAc_2\ Hex_5\ HexNAc_5$	3037.5	0.16
$Fuc_3\ Hex_6\ HexNAc_5$	3041.6	0.43
$NeuAc_1\ Fuc_1\ Hex_6\ HexNAc_5$	3054.5	3.12
$Fuc_2\ Hex_6\ HexNAc_6$	3112.6	0.07
$NeuAc_1\ Hex_6\ HexNAc_6$	3125.6	0.05
$NeuAc_2\ Fuc_2\ Hex_5\ HexNAc_4$	3140.6	0.11
$Fuc_1\ Hex_7\ HexNAc_6$	3142.6	0.94
$Fuc_1\ Hex_6\ HexNAc_7$	3183.6	0.04
$NeuAc_1\ Fuc_3\ Hex_5\ HexNAc_5$	3198.6	0.09
$NeuAc_2\ Fuc_1\ Hex_5\ HexNAc_5$	3211.6	0.98
$Hex_7\ HexNAc_7$	3213.6	1.36
$NeuAc_1\ Fuc_2\ Hex_6\ HexNAc_5$	3227.6	0.99
$NeuAc_2\ Hex_6\ HexNAc_5$	3241.6	2.11
$Fuc_3\ Hex_6\ HexNAc_6$	3286.7	0.06
$NeuAc_1\ Fuc_1\ Hex_6\ HexNAc_6$	3299.7	0.18
$NeuAc_1\ Hex_7\ HexNAc_7$	3314.7	0.01
$Fuc_2\ Hex_7\ HexNAc_6$	3316.6	0.14
$NeuAc_3\ Fuc_2\ Hex_5\ HexNAc_4$	3327.7	0.06
$NeuAc_1\ Fuc_1\ Hex_7\ HexNAc_6$	3329.7	0.26
$NeuAc_2\ Fuc_2\ Hex_5\ HexNAc_5$	3385.7	0.02
$Fuc_1\ Hex_7\ HexNAc_7$	3387.7	0.35
$NeuAc_1\ Fuc_3\ Hex_6\ HexNAc_5$	3402.7	0.71
$NeuAc_2\ Fuc_1\ Hex_6\ HexNAc_5$	3415.8	2.86
$NeuAc_1\ Fuc_2\ Hex_6\ HexNAc_6$	3473.7	0.10
$NeuAc_2\ Hex_6\ HexNAc_6$	3486.8	0.04
$NeuAc_2\ Fuc_4\ Hex_5\ HexNAc_4$	3488.8	0.06
$Fuc_3\ Hex_7\ HexNAc_6$	3490.8	0.18
$NeuAc_1\ Fuc_1\ Hex_7\ HexNAc_6$	3503.8	1.33
$NeuAc_2\ Fuc_2\ Hex_5\ HexNAc_5$	3559.8	0.02
$Fuc_2\ Hex_7\ HexNAc_7$	3561.8	0.07
$NeuAc_1\ Hex_7\ HexNAc_7$	3574.8	0.10
$NeuAc_2\ Fuc_2\ Hex_6\ HexNAc_5$	3589.8	0.60
$Fuc_1\ Hex_8\ HexNAc_7$	3591.8	0.88

$NeuAc_3 Hex_6 HexNAc_5$	3602.8	16.31
$Fuc_1 Hex_7 HexNAc_8$	3632.8	0.08
$NeuAc_1 Fuc_3 Hex_6 HexNAc_6$	3647.8	0.04
$NeuAc_2 Fuc_1 Hex_6 HexNAc_6$	3660.8	0.11
$Hex_8 HexNAc_8$	3662.8	0.42
$NeuAc_1 Fuc_2 Hex_7 HexNAc_6$	3677.8	0.77
$NeuAc_2 Hex_7 HexNAc_6$	3690.9	0.63
$Fuc_3 Hex_7 HexNAc_7$	3735.9	0.03
$NeuAc_1 Fuc_1 Hex_7 HexNAc_7$	3748.9	0.09
$NeuAc_2 Fuc_3 Hex_6 HexNAc_5$	3763.9	0.07
$NeuAc_3 Fuc_1 Hex_6 HexNAc_5$	3776.9	2.19
$Fuc_2 Hex_7 HexNAc_8$	3806.9	0.04
$NeuAc_2 Fuc_2 Hex_6 HexNAc_6$	3834.9	0.03
$Fuc_1 Hex_8 HexNAc_8$	3836.9	0.19
$NeuAc_3 Hex_6 HexNAc_6$	3847.9	0.02
$NeuAc_1 Fuc_3 Hex_7 HexNAc_6$	3851.9	0.47
$NeuAc_2 Fuc_1 Hex_7 HexNAc_6$	3864.9	0.81
$Fuc_4 Hex_7 HexNAc_7$	3910.0	0.04
$NeuAc_2 Hex_7 HexNAc_7$	3936.0	0.01
$NeuAc_2 Fuc_4 Hex_6 HexNAc_5$	3938.0	0.03
$Fuc_3 Hex_8 HexNAc_7$	3940.0	0.08
$NeuAc_3 Fuc_2 Hex_6 HexNAc_5$	3951.0	0.04
$NeuAc_1 Fuc_1 Hex_8 HexNAc_7$	3953.0	0.46
$NeuAc_2 Fuc_3 Hex_6 HexNAc_6$	4009.0	0.01
$Fuc_2 Hex_8 HexNAc_8$	4011.0	0.03
$NeuAc_3 Fuc_1 Hex_6 HexNAc_6$	4022.0	0.01
$NeuAc_1 Hex_8 HexNAc_8$	4024.0	0.07
$NeuAc_1 Fuc_4 Hex_7 HexNAc_6$	4026.0	0.12
$NeuAc_2 Fuc_2 Hex_7 HexNAc_6$	4039.0	0.51
$NeuAc_3 Hex_7 HexNAc_6$	4052.0	1.65
$NeuAc_3 Fuc_3 Hex_6 HexNAc_5$	4125.0	0.02
$NeuAc_1 Fuc_2 Hex_8 HexNAc_7$	4127.0	0.20
$NeuAc_4 Fuc_1 Hex_6 HexNAc_5$	4138.1	0.04
$NeuAc_2 Hex_8 HexNAc_7$	4140.1	0.16
$NeuAc_2 Fuc_4 Hex_6 HexNAc_6$	4183.1	0.01
$Fuc_3 Hex_8 HexNAc_8$	4185.1	0.03
$NeuAc_1 Fuc_1 Hex_8 HexNAc_8$	4198.1	0.06
$NeuAc_2 Fuc_3 Hex_7 HexNAc_6$	4213.1	0.15
$NeuAc_3 Fuc_1 Hex_7 HexNAc_6$	4226.1	0.33
$NeuAc_2 Fuc_2 Hex_7 HexNAc_7$	4284.1	0.02
$Fuc_1 Hex_9 HexNAc_9$	4286.1	0.05
$NeuAc_3 Hex_7 HexNAc_7$	4297.2	0.01
$NeuAc_3 Fuc_4 Hex_6 HexNAc_5$	4299.2	0.02
$NeuAc_1 Fuc_3 Hex_8 HexNAc_7$	4301.2	0.21
$NeuAc_2 Fuc_1 Hex_8 HexNAc_7$	4314.2	0.25

$NeuAc_2\ Hex_8\ HexNAc_8$	4385.2	0.02
$NeuAc_2\ Fuc_4\ Hex_7\ HexNAc_6$	4387.2	0.04
$Fuc_3\ Hex_9\ HexNAc_8$	4389.2	0.06
$NeuAc_3\ Fuc_2\ Hex_7\ HexNAc_6$	4400.2	0.11
$NeuAc_1\ Fuc_1\ Hex_9\ HexNAc_8$	4402.2	0.65
$NeuAc_4\ Hex_7\ HexNAc_6$	4413.2	2.55
$NeuAc_3\ Fuc_1\ Hex_7\ HexNAc_7$	4471.2	0.01
$NeuAc_1\ Hex_9\ HexNAc_9$	4473.2	0.04
$NeuAc_1\ Fuc_4\ Hex_8\ HexNAc_7$	4475.2	0.11
$NeuAc_2\ Fuc_2\ Hex_8\ HexNAc_7$	4488.2	0.11
$NeuAc_3\ Hex_8\ HexNAc_7$	4501.2	0.07
$NeuAc_3\ Fuc_3\ Hex_7\ HexNAc_6$	4574.2	0.03
$NeuAc_1\ Fuc_2\ Hex_9\ HexNAc_8$	4576.3	0.10
$NeuAc_4\ Fuc_1\ Hex_7\ HexNAc_6$	4587.3	0.26
$NeuAc_2\ Fuc_4\ Hex_7\ HexNAc_7$	4632.3	<0.01
$Fuc_3\ Hex_9\ HexNAc_9$	4634.3	0.01
$NeuAc_3\ Fuc_2\ Hex_7\ HexNAc_7$	4645.3	<0.01
$NeuAc_1\ Fuc_1\ Hex_9\ HexNAc_9$	4647.3	0.02
$NeuAc_1\ Fuc_5\ Hex_8\ HexNAc_7$	4649.3	0.07
$NeuAc_2\ Fuc_3\ Hex_8\ HexNAc_7$	4662.3	0.07
$NeuAc_3\ Fuc_1\ Hex_8\ HexNAc_7$	4675.4	0.09
$NeuAc_1\ Fuc_4\ Hex_8\ HexNAc_8$	4720.4	0.01
$NeuAc_2\ Fuc_2\ Hex_8\ HexNAc_8$	4733.4	0.01
$Fuc_1\ Hex_{10}\ HexNAc_{10}$	4735.4	0.03
$NeuAc_3\ Hex_8\ HexNAc_8$	4746.4	<0.01
$NeuAc_3\ Fuc_4\ Hex_7\ HexNAc_6$	4748.4	0.01
$NeuAc_1\ Fuc_3\ Hex_9\ HexNAc_8$	4750.4	0.11
$NeuAc_4\ Fuc_2\ Hex_7\ HexNAc_6$	4761.4	0.01
$NeuAc_2\ Fuc_1\ Hex_9\ HexNAc_8$	4763.5	0.15
$NeuAc_2\ Fuc_5\ Hex_7\ HexNAc_7$	4806.4	0.01
$Fuc_4\ Hex_9\ HexNAc_9$	4808.4	0.01
$NeuAc_3\ Fuc_3\ Hex_7\ HexNAc_7$	4819.4	<0.01
$NeuAc_1\ Fuc_2\ Hex_9\ HexNAc_9$	4821.4	0.01
$NeuAc_1\ Fuc_6\ Hex_8\ HexNAc_7$	4823.4	0.01
$NeuAc_2\ Hex_9\ HexNAc_9$	4834.4	<0.01
$NeuAc_2\ Fuc_4\ Hex_8\ HexNAc_7$	4836.4	0.02
$NeuAc_3\ Fuc_2\ Hex_8\ HexNAc_7$	4849.4	0.03
$NeuAc_1\ Fuc_1\ Hex_{10}\ HexNAc_9$	4851.4	0.10
$NeuAc_4\ Hex_8\ HexNAc_7$	4862.4	0.06
$NeuAc_1\ Fuc_5\ Hex_8\ HexNAc_8$	4894.5	<0.01
$NeuAc_2\ Fuc_3\ Hex_8\ HexNAc_8$	4907.5	<0.01
$Fuc_2\ Hex_{10}\ HexNAc_{10}$	4909.5	0.01
$NeuAc_3\ Fuc_1\ Hex_8\ HexNAc_8$	4920.5	0.01
$NeuAc_1\ Hex_{10}\ HexNAc_{10}$	4922.5	0.02
$NeuAc_1\ Fuc_4\ Hex_9\ HexNAc_8$	4924.5	0.09

$NeuAc_2$ Fuc_2 Hex_9 $HexNAc_8$	4937.5	0.06
$NeuAc_5$ Fuc_1 Hex_7 $HexNAc_6$	4948.5	0.01
$NeuAc_3$ Hex_9 $HexNAc_8$	4950.5	0.06
$NeuAc_2$ Fuc_6 Hex_7 $HexNAc_7$	4980.5	<0.01
$NeuAc_3$ Fuc_4 Hex_7 $HexNAc_7$	4993.5	<0.01
$NeuAc_1$ Fuc_3 Hex_9 $HexNAc_9$	4995.5	0.01
$NeuAc_2$ Fuc_1 Hex_9 $HexNAc_9$	5008.6	<0.01
$NeuAc_2$ Fuc_5 Hex_8 $HexNAc_7$	5010.6	0.03
$NeuAc_3$ Fuc_3 Hex_8 $HexNAc_7$	5023.6	0.01
$NeuAc_1$ Fuc_2 Hex_{10} $HexNAc_9$	5025.6	0.04
$NeuAc_4$ Fuc_1 Hex_8 $HexNAc_7$	5036.6	0.03
$NeuAc_1$ Fuc_6 Hex_8 $HexNAc_8$	5068.6	<0.01
$NeuAc_2$ Fuc_4 Hex_8 $HexNAc_8$	5081.6	<0.01
Fuc_3 Hex_{10} $HexNAc_{10}$	5083.6	0.01
$NeuAc_3$ Fuc_2 Hex_8 $HexNAc_8$	5094.6	<0.01
$NeuAc_1$ Fuc_1 Hex_{10} $HexNAc_{10}$	5096.6	0.01
$NeuAc_1$ Fuc_5 Hex_9 $HexNAc_8$	5098.6	0.04
$NeuAc_2$ Fuc_3 Hex_9 $HexNAc_8$	5111.6	0.03
$NeuAc_3$ Fuc_1 Hex_9 $HexNAc_8$	5124.6	0.03
$NeuAc_1$ Fuc_4 Hex_9 $HexNAc_9$	5169.7	<0.01
$NeuAc_2$ Fuc_2 Hex_9 $HexNAc_9$	5182.7	<0.01
$NeuAc_2$ Fuc_6 Hex_8 $HexNAc_7$	5184.7	0.01
$NeuAc_3$ Hex_9 $HexNAc_9$	5195.7	<0.01
$NeuAc_3$ Fuc_4 Hex_8 $HexNAc_7$	5197.7	0.01
$NeuAc_1$ Fuc_3 Hex_{10} $HexNAc_9$	5199.7	0.03
$NeuAc_4$ Fuc_2 Hex_8 $HexNAc_7$	5210.7	<0.01
$NeuAc_2$ Fuc_1 Hex_{10} $HexNAc_9$	5212.7	0.04
$NeuAc_2$ Fuc_5 Hex_8 $HexNAc_8$	5255.7	<0.01
$NeuAc_3$ Fuc_3 Hex_8 $HexNAc_8$	5268.7	<0.01
$NeuAc_1$ Fuc_2 Hex_{10} $HexNAc_{10}$	5270.7	<0.01
$NeuAc_1$ Fuc_6 Hex_9 $HexNAc_8$	5272.7	0.01
$NeuAc_2$ Hex_{10} $HexNAc_{10}$	5283.8	<0.01
$NeuAc_2$ Fuc_4 Hex_9 $HexNAc_8$	5285.8	0.02
$NeuAc_3$ Fuc_2 Hex_9 $HexNAc_8$	5298.8	<0.01
$NeuAc_1$ Fuc_1 Hex_{11} $HexNAc_{10}$	5300.8	0.04
$NeuAc_4$ Hex_9 $HexNAc_8$	5311.8	0.01
$NeuAc_1$ Fuc_5 Hex_9 $HexNAc_9$	5343.8	<0.01
$NeuAc_2$ Fuc_3 Hex_9 $HexNAc_9$	5356.8	<0.01
Fuc_2 Hex_{11} $HexNAc_{11}$	5358.8	0.01
$NeuAc_3$ Fuc_1 Hex_9 $HexNAc_9$	5369.8	<0.01
$NeuAc_1$ Hex_{11} $HexNAc_{11}$	5371.8	0.01
$NeuAc_1$ Fuc_4 Hex_{10} $HexNAc_9$	5373.8	0.02
$NeuAc_4$ Fuc_3 Hex_8 $HexNAc_7$	5384.8	<0.01
$NeuAc_2$ Fuc_2 Hex_{10} $HexNAc_9$	5386.8	0.01
$NeuAc_5$ Fuc_1 Hex_8 $HexNAc_7$	5397.8	<0.01

$NeuAc_3$ Hex_{10} $HexNAc_9$	5399.8	0.01
$NeuAc_2$ Fuc_6 Hex_8 $HexNAc_8$	5429.8	<0.01
$NeuAc_3$ Fuc_4 Hex_8 $HexNAc_8$	5442.8	<0.01
$NeuAc_1$ Fuc_3 Hex_{10} $HexNAc_{10}$	5444.8	<0.01
$NeuAc_1$ Fuc_7 Hex_9 $HexNAc_8$	5446.8	<0.01
$NeuAc_2$ Fuc_1 Hex_{10} $HexNAc_{10}$	5457.8	<0.01
$NeuAc_2$ Fuc_5 Hex_9 $HexNAc_8$	5459.8	0.02
$NeuAc_3$ Fuc_3 Hex_9 $HexNAc_8$	5472.8	0.01
$NeuAc_1$ Fuc_2 Hex_{11} $HexNAc_{10}$	5474.8	0.02
$NeuAc_4$ Fuc_1 Hex_9 $HexNAc_8$	5485.8	0.01
$NeuAc_2$ Fuc_4 Hex_9 $HexNAc_9$	5530.9	<0.01
Fuc_3 Hex_{11} $HexNAc_{11}$	5532.9	<0.01
$NeuAc_1$ Fuc_1 Hex_{11} $HexNAc_{11}$	5545.9	0.01
$NeuAc_1$ Fuc_5 Hex_{10} $HexNAc_9$	5547.9	0.02
$NeuAc_4$ Fuc_4 Hex_8 $HexNAc_7$	5558.9	<0.01
$NeuAc_2$ Fuc_3 Hex_{10} $HexNAc_9$	5560.9	0.01
$NeuAc_3$ Fuc_1 Hex_{10} $HexNAc_9$	5573.9	0.01
$NeuAc_1$ Fuc_4 Hex_{10} $HexNAc_{10}$	5618.9	<0.01
$NeuAc_2$ Fuc_2 Hex_{10} $HexNAc_{10}$	5631.9	<0.01
$NeuAc_2$ Fuc_6 Hex_9 $HexNAc_8$	5633.9	<0.01
$NeuAc_3$ Fuc_4 Hex_9 $HexNAc_8$	5646.9	<0.01
$NeuAc_1$ Fuc_3 Hex_{11} $HexNAc_{10}$	5648.9	0.01
$NeuAc_4$ Fuc_2 Hex_9 $HexNAc_8$	5659.9	<0.01
$NeuAc_2$ Fuc_1 Hex_{11} $HexNAc_{10}$	5661.9	0.03
$NeuAc_5$ Hex_9 $HexNAc_8$	5673.0	0.01
$NeuAc_1$ Fuc_2 Hex_{11} $HexNAc_{11}$	5720.0	<0.01
$NeuAc_1$ Fuc_6 Hex_{10} $HexNAc_9$	5722.0	<0.01
$NeuAc_2$ Hex_{11} $HexNAc_{11}$	5733.0	<0.01
$NeuAc_2$ Fuc_4 Hex_{10} $HexNAc_9$	5735.0	0.01
$NeuAc_3$ Fuc_2 Hex_{10} $HexNAc_9$	5748.0	0.01
$NeuAc_1$ Fuc_1 Hex_{12} $HexNAc_{11}$	5750.0	0.02
$NeuAc_4$ Hex_{10} $HexNAc_9$	5761.0	<0.01
$NeuAc_3$ Fuc_1 Hex_{10} $HexNAc_{10}$	5819.0	<0.01
$NeuAc_1$ Hex_{12} $HexNAc_{12}$	5821.0	0.01
$NeuAc_1$ Fuc_4 Hex_{11} $HexNAc_{10}$	5823.0	0.01
$NeuAc_4$ Fuc_3 Hex_9 $HexNAc_8$	5834.0	<0.01
$NeuAc_2$ Fuc_2 Hex_{11} $HexNAc_{10}$	5836.0	0.01
Fuc_1 Hex_{13} $HexNAc_{12}$	5838.0	0.01
$NeuAc_5$ Fuc_1 Hex_9 $HexNAc_8$	5847.0	0.01
$NeuAc_1$ Fuc_3 Hex_{11} $HexNAc_{11}$	5894.0	<0.01
$NeuAc_2$ Fuc_1 Hex_{11} $HexNAc_{11}$	5907.1	<0.01
$NeuAc_2$ Fuc_5 Hex_{10} $HexNAc_9$	5909.1	0.01
$NeuAc_3$ Fuc_3 Hex_{10} $HexNAc_9$	5922.1	<0.01
$NeuAc_1$ Fuc_2 Hex_{12} $HexNAc_{11}$	5924.1	0.01
$NeuAc_4$ Fuc_1 Hex_{10} $HexNAc_9$	5935.1	<0.01

NeuAc$_1$ Fuc$_6$ Hex$_{10}$ HexNAc$_{10}$	5967.1	<0.01
NeuAc$_1$ Fuc$_1$ Hex$_{12}$ HexNAc$_{12}$	5995.2	<0.01
NeuAc$_1$ Fuc$_5$ Hex$_{11}$ HexNAc$_{10}$	5997.2	0.01
NeuAc$_4$ Fuc$_4$ Hex$_9$ HexNAc$_8$	6008.2	<0.01
NeuAc$_2$ Fuc$_3$ Hex$_{11}$ HexNAc$_{10}$	6010.2	0.01
NeuAc$_5$ Fuc$_2$ Hex$_9$ HexNAc$_8$	6021.2	<0.01
NeuAc$_3$ Fuc$_1$ Hex$_{11}$ HexNAc$_{10}$	6023.2	0.01
NeuAc$_2$ Fuc$_6$ Hex$_{10}$ HexNAc$_9$	6083.2	<0.01
NeuAc$_3$ Fuc$_4$ Hex$_{10}$ HexNAc$_9$	6096.3	<0.01
NeuAc$_1$ Fuc$_3$ Hex$_{12}$ HexNAc$_{11}$	6098.3	<0.01
NeuAc$_4$ Fuc$_2$ Hex$_{10}$ HexNAc$_9$	6109.3	<0.01
NeuAc$_2$ Fuc$_1$ Hex$_{12}$ HexNAc$_{11}$	6111.3	0.01
NeuAc$_1$ Fuc$_2$ Hex$_{12}$ HexNAc$_{12}$	6169.3	<0.01
NeuAc$_1$ Fuc$_6$ Hex$_{11}$ HexNAc$_{10}$	6171.3	<0.01
NeuAc$_2$ Hex$_{12}$ HexNAc$_{12}$	6182.3	<0.01
NeuAc$_2$ Fuc$_4$ Hex$_{11}$ HexNAc$_{10}$	6184.3	0.01
NeuAc$_3$ Fuc$_2$ Hex$_{11}$ HexNAc$_{10}$	6197.4	<0.01
NeuAc$_1$ Fuc$_1$ Hex$_{13}$ HexNAc$_{12}$	6199.4	<0.01
NeuAc$_1$ Hex$_{13}$ HexNAc$_{13}$	6270.5	<0.01
NeuAc$_1$ Fuc$_4$ Hex$_{12}$ HexNAc$_{11}$	6272.5	<0.01
NeuAc$_2$ Fuc$_2$ Hex$_{12}$ HexNAc$_{11}$	6285.5	<0.01
NeuAc$_2$ Fuc$_5$ Hex$_{11}$ HexNAc$_{10}$	6358.6	<0.01
NeuAc$_1$ Fuc$_2$ Hex$_{13}$ HexNAc$_{12}$	6373.6	<0.01
NeuAc$_4$ Fuc$_1$ Hex$_{11}$ HexNAc$_{10}$	6384.6	<0.01
NeuAc$_1$ Fuc$_5$ Hex$_{12}$ HexNAc$_{11}$	6446.7	<0.01
NeuAc$_2$ Fuc$_3$ Hex$_{12}$ HexNAc$_{11}$	6459.7	<0.01
NeuAc$_3$ Fuc$_1$ Hex$_{12}$ HexNAc$_{11}$	6472.7	<0.01
NeuAc$_1$ Fuc$_3$ Hex$_{13}$ HexNAc$_{12}$	6547.7	<0.01
NeuAc$_2$ Fuc$_1$ Hex$_{13}$ HexNAc$_{12}$	6560.7	<0.01
NeuAc$_3$ Fuc$_2$ Hex$_{12}$ HexNAc$_{11}$	6646.7	<0.01
NeuAc$_1$ Fuc$_1$ Hex$_{14}$ HexNAc$_{13}$	6648.7	<0.01
NeuAc$_1$ Hex$_{14}$ HexNAc$_{14}$	6719.8	<0.01
NeuAc$_1$ Fuc$_4$ Hex$_{13}$ HexNAc$_{12}$	6721.8	<0.01
NeuAc$_2$ Fuc$_2$ Hex$_{13}$ HexNAc$_{12}$	6734.8	<0.01
NeuAc$_1$ Fuc$_2$ Hex$_{14}$ HexNAc$_{13}$	6822.8	<0.01
NeuAc$_1$ Fuc$_5$ Hex$_{13}$ HexNAc$_{12}$	6895.8	<0.01
NeuAc$_3$ Fuc$_1$ Hex$_{13}$ HexNAc$_{12}$	6921.8	<0.01

PNGase F released N-glycan (5000-12000)

Composition	Observed average Mass (m/z)	Relative abundance (%)
NeuAc$_2$ Fuc$_5$ Hex$_8$ HexNAc$_7$	5013.1	29.87
NeuAc$_1$ Fuc$_2$ Hex$_{10}$ HexNAc$_9$	5027.7	47.30
NeuAc$_4$ Fuc$_1$ Hex$_8$ HexNAc$_7$	5040.1	100.00
NeuAc$_1$ Fuc$_6$ Hex$_8$ HexNAc$_8$	5071.1	6.01

$NeuAc_2\ Fuc_4\ Hex_8\ HexNAc_8$	5086.2	12.18
$NeuAc_1\ Fuc_5\ Hex_9\ HexNAc_8$	5100.9	59.63
$NeuAc_2\ Fuc_3\ Hex_9\ HexNAc_8$	5114.8	82.64
$NeuAc_3\ Fuc_1\ Hex_9\ HexNAc_8$	5128.1	94.46
$NeuAc_1\ Fuc_4\ Hex_9\ HexNAc_9$	5173.6	12.77
$NeuAc_2\ Fuc_6\ Hex_8\ HexNAc_7$	5188.3	20.63
$NeuAc_1\ Fuc_3\ Hex_{10}\ HexNAc_9$	5202.4	46.90
$NeuAc_2\ Fuc_1\ Hex_{10}\ HexNAc_9$	5215.8	89.16
$NeuAc_2\ Fuc_5\ Hex_8\ HexNAc_8$	5260.1	6.66
$NeuAc_1\ Fuc_6\ Hex_9\ HexNAc_8$	5275.2	14.87
$NeuAc_2\ Fuc_4\ Hex_9\ HexNAc_8$	5289.1	44.11
$NeuAc_1\ Fuc_1\ Hex_{11}\ HexNAc_{10}$	5302.8	66.46
$NeuAc_4\ Hex_9\ HexNAc_8$	5315.3	45.12
$NeuAc_1\ Fuc_5\ Hex_9\ HexNAc_9$	5347.3	5.68
$NeuAc_2\ Fuc_3\ Hex_9\ HexNAc_9$	5361.9	12.51
$NeuAc_1\ Fuc_4\ Hex_{10}\ HexNAc_9$	5376.4	39.51
$NeuAc_2\ Fuc_2\ Hex_{10}\ HexNAc_9$	5390.3	40.14
$NeuAc_3\ Hex_{10}\ HexNAc_9$	5402.8	14.67
$NeuAc_2\ Fuc_6\ Hex_8\ HexNAc_8$	5433.8	4.91
$NeuAc_1\ Fuc_3\ Hex_{10}\ HexNAc_{10}$	5448.2	7.66
$NeuAc_2\ Fuc_5\ Hex_9\ HexNAc_8$	5462.6	24.25
$NeuAc_1\ Fuc_2\ Hex_{11}\ HexNAc_{10}$	5476.8	35.61
$NeuAc_4\ Fuc_1\ Hex_9\ HexNAc_8$	5489.7	65.38
$NeuAc_1\ Fuc_6\ Hex_9\ HexNAc_9$	5520.7	4.04
$NeuAc_2\ Fuc_4\ Hex_9\ HexNAc_9$	5535.7	10.52
$NeuAc_1\ Fuc_5\ Hex_{10}\ HexNAc_9$	5550.3	31.57
$NeuAc_2\ Fuc_3\ Hex_{10}\ HexNAc_9$	5564.3	37.41
$NeuAc_3\ Fuc_1\ Hex_{10}\ HexNAc_9$	5577.7	45.83
$NeuAc_1\ Fuc_4\ Hex_{10}\ HexNAc_{10}$	5622.6	6.06
$NeuAc_2\ Fuc_6\ Hex_9\ HexNAc_8$	5637.7	11.57
$NeuAc_1\ Fuc_3\ Hex_{11}\ HexNAc_{10}$	5651.6	25.17
$NeuAc_2\ Fuc_1\ Hex_{11}\ HexNAc_{10}$	5665.1	53.08
$NeuAc_2\ Fuc_5\ Hex_9\ HexNAc_9$	5709.5	4.77
$NeuAc_1\ Fuc_6\ Hex_{10}\ HexNAc_9$	5724.6	10.51
$NeuAc_2\ Fuc_4\ Hex_{10}\ HexNAc_9$	5738.5	24.40
$NeuAc_1\ Fuc_1\ Hex_{12}\ HexNAc_{11}$	5752.5	31.07
$NeuAc_4\ Hex_{10}\ HexNAc_9$	5765.0	9.64
$NeuAc_1\ Fuc_5\ Hex_{10}\ HexNAc_{10}$	5796.4	3.67
$NeuAc_2\ Fuc_3\ Hex_{10}\ HexNAc_{10}$	5811.0	6.18
$NeuAc_1\ Fuc_4\ Hex_{11}\ HexNAc_{10}$	5825.5	19.21
$NeuAc_2\ Fuc_2\ Hex_{11}\ HexNAc_{10}$	5839.3	23.07
$NeuAc_3\ Hex_{11}\ HexNAc_{10}$	5851.1	40.52
$NeuAc_2\ Fuc_6\ Hex_9\ HexNAc_9$	5883.0	2.97
$NeuAc_1\ Fuc_3\ Hex_{11}\ HexNAc_{11}$	5898.0	5.00
$NeuAc_2\ Fuc_5\ Hex_{10}\ HexNAc_9$	5912.1	16.32

NeuAc$_1$ Fuc$_2$ Hex$_{12}$ HexNAc$_{11}$	5926.3	17.95
NeuAc$_4$ Fuc$_1$ Hex$_{10}$ HexNAc$_9$	5939.3	25.86
NeuAc$_1$ Fuc$_6$ Hex$_{10}$ HexNAc$_{10}$	5970.0	2.33
NeuAc$_2$ Fuc$_4$ Hex$_{10}$ HexNAc$_{10}$	5985.2	4.82
NeuAc$_1$ Fuc$_5$ Hex$_{11}$ HexNAc$_{10}$	5999.8	15.32
NeuAc$_2$ Fuc$_3$ Hex$_{11}$ HexNAc$_{10}$	6013.6	19.75
NeuAc$_3$ Fuc$_1$ Hex$_{11}$ HexNAc$_{10}$	6026.8	30.77
NeuAc$_1$ Fuc$_4$ Hex$_{11}$ HexNAc$_{11}$	6072.1	3.43
NeuAc$_2$ Fuc$_6$ Hex$_{10}$ HexNAc$_9$	6087.1	7.46
NeuAc$_1$ Fuc$_3$ Hex$_{12}$ HexNAc$_{11}$	6100.9	14.37
NeuAc$_2$ Fuc$_1$ Hex$_{12}$ HexNAc$_{11}$	6114.7	27.87
NeuAc$_2$ Fuc$_5$ Hex$_{10}$ HexNAc$_{10}$	6159.2	2.88
NeuAc$_1$ Fuc$_6$ Hex$_{11}$ HexNAc$_{10}$	6174.1	6.34
NeuAc$_2$ Fuc$_4$ Hex$_{11}$ HexNAc$_{10}$	6187.8	13.37
NeuAc$_1$ Fuc$_1$ Hex$_{13}$ HexNAc$_{12}$	6202.1	16.80
NeuAc$_4$ Hex$_{11}$ HexNAc$_{10}$	6214.9	2.28
NeuAc$_1$ Fuc$_5$ Hex$_{11}$ HexNAc$_{11}$	6246.1	1.96
NeuAc$_2$ Fuc$_3$ Hex$_{11}$ HexNAc$_{11}$	6260.6	3.52
NeuAc$_1$ Fuc$_4$ Hex$_{12}$ HexNAc$_{11}$	6274.8	10.70
NeuAc$_2$ Fuc$_2$ Hex$_{12}$ HexNAc$_{11}$	6289.0	12.41
NeuAc$_3$ Hex$_{12}$ HexNAc$_{11}$	6301.2	8.96
NeuAc$_2$ Fuc$_6$ Hex$_{10}$ HexNAc$_{10}$	6332.8	1.77
NeuAc$_1$ Fuc$_3$ Hex$_{12}$ HexNAc$_{12}$	6347.5	3.90
NeuAc$_2$ Fuc$_5$ Hex$_{11}$ HexNAc$_{10}$	6361.7	11.12
NeuAc$_1$ Fuc$_2$ Hex$_{13}$ HexNAc$_{12}$	6375.7	10.53
NeuAc$_4$ Fuc$_1$ Hex$_{11}$ HexNAc$_{10}$	6388.9	13.97
NeuAc$_1$ Fuc$_6$ Hex$_{11}$ HexNAc$_{11}$	6419.7	1.62
NeuAc$_2$ Fuc$_4$ Hex$_{11}$ HexNAc$_{11}$	6434.6	3.04
NeuAc$_1$ Fuc$_5$ Hex$_{12}$ HexNAc$_{11}$	6449.1	8.11
NeuAc$_2$ Fuc$_3$ Hex$_{12}$ HexNAc$_{11}$	6463.2	10.82
NeuAc$_3$ Fuc$_1$ Hex$_{12}$ HexNAc$_{11}$	6476.5	16.29
NeuAc$_1$ Fuc$_4$ Hex$_{12}$ HexNAc$_{12}$	6521.6	2.27
NeuAc$_2$ Fuc$_6$ Hex$_{11}$ HexNAc$_{10}$	6536.4	5.12
NeuAc$_1$ Fuc$_3$ Hex$_{13}$ HexNAc$_{12}$	6550.4	9.00
NeuAc$_2$ Fuc$_1$ Hex$_{13}$ HexNAc$_{12}$	6564.2	15.45
NeuAc$_2$ Fuc$_5$ Hex$_{11}$ HexNAc$_{11}$	6608.5	1.80
NeuAc$_1$ Fuc$_6$ Hex$_{12}$ HexNAc$_{11}$	6623.5	3.70
NeuAc$_2$ Fuc$_4$ Hex$_{12}$ HexNAc$_{11}$	6637.2	7.34
NeuAc$_1$ Fuc$_1$ Hex$_{14}$ HexNAc$_{13}$	6651.5	9.22
NeuAc$_4$ Hex$_{12}$ HexNAc$_{11}$	6664.2	1.10
NeuAc$_1$ Fuc$_5$ Hex$_{12}$ HexNAc$_{12}$	6695.2	1.20
NeuAc$_2$ Fuc$_3$ Hex$_{12}$ HexNAc$_{12}$	6710.0	2.35
NeuAc$_1$ Fuc$_4$ Hex$_{13}$ HexNAc$_{12}$	6724.1	6.53
NeuAc$_2$ Fuc$_2$ Hex$_{13}$ HexNAc$_{12}$	6738.5	7.39
NeuAc$_3$ Hex$_{13}$ HexNAc$_{12}$	6750.8	5.29

$NeuAc_2\ Fuc_6\ Hex_{11}\ HexNAc_{11}$	6782.2	1.24
$NeuAc_1\ Fuc_3\ Hex_{13}\ HexNAc_{13}$	6797.1	2.66
$NeuAc_2\ Fuc_5\ Hex_{12}\ HexNAc_{11}$	6811.1	6.54
$NeuAc_1\ Fuc_2\ Hex_{14}\ HexNAc_{13}$	6825.1	5.87
$NeuAc_4\ Fuc_1\ Hex_{12}\ HexNAc_{11}$	6838.6	7.10
$NeuAc_1\ Fuc_6\ Hex_{12}\ HexNAc_{12}$	6869.2	0.88
$NeuAc_2\ Fuc_4\ Hex_{12}\ HexNAc_{12}$	6884.2	1.85
$NeuAc_1\ Fuc_5\ Hex_{13}\ HexNAc_{12}$	6898.5	4.53
$NeuAc_2\ Fuc_3\ Hex_{13}\ HexNAc_{12}$	6912.6	6.27
$NeuAc_3\ Fuc_1\ Hex_{13}\ HexNAc_{12}$	6926.0	9.86
$NeuAc_1\ Fuc_4\ Hex_{13}\ HexNAc_{13}$	6970.9	1.59
$NeuAc_2\ Fuc_6\ Hex_{12}\ HexNAc_{11}$	6985.9	3.41
$NeuAc_1\ Fuc_3\ Hex_{14}\ HexNAc_{13}$	6999.8	5.41
$NeuAc_2\ Fuc_1\ Hex_{14}\ HexNAc_{13}$	7013.8	8.38
$NeuAc_2\ Fuc_5\ Hex_{12}\ HexNAc_{12}$	7057.8	1.11
$NeuAc_1\ Fuc_6\ Hex_{13}\ HexNAc_{12}$	7072.7	2.04
$NeuAc_2\ Fuc_4\ Hex_{13}\ HexNAc_{12}$	7086.5	4.26
$NeuAc_1\ Fuc_1\ Hex_{15}\ HexNAc_{14}$	7101.0	5.26
$NeuAc_4\ Hex_{13}\ HexNAc_{12}$	7113.7	0.96
$NeuAc_1\ Fuc_5\ Hex_{13}\ HexNAc_{13}$	7144.4	0.85
$NeuAc_2\ Fuc_3\ Hex_{13}\ HexNAc_{13}$	7159.3	1.67
$NeuAc_1\ Fuc_4\ Hex_{14}\ HexNAc_{13}$	7173.4	4.21
$NeuAc_2\ Fuc_2\ Hex_{14}\ HexNAc_{13}$	7187.9	3.95
$NeuAc_3\ Hex_{14}\ HexNAc_{13}$	7200.8	2.41
$NeuAc_2\ Fuc_6\ Hex_{12}\ HexNAc_{12}$	7231.7	0.67
$NeuAc_1\ Fuc_3\ Hex_{14}\ HexNAc_{14}$	7246.4	1.60
$NeuAc_2\ Fuc_5\ Hex_{13}\ HexNAc_{12}$	7260.6	4.00
$NeuAc_1\ Fuc_2\ Hex_{15}\ HexNAc_{14}$	7274.5	3.35
$NeuAc_4\ Fuc_1\ Hex_{13}\ HexNAc_{12}$	7288.0	3.95
$NeuAc_1\ Fuc_6\ Hex_{13}\ HexNAc_{13}$	7318.4	0.59
$NeuAc_2\ Fuc_4\ Hex_{13}\ HexNAc_{13}$	7333.3	1.19
$NeuAc_1\ Fuc_5\ Hex_{14}\ HexNAc_{13}$	7347.8	2.74
$NeuAc_2\ Fuc_3\ Hex_{14}\ HexNAc_{13}$	7362.1	3.54
$NeuAc_3\ Fuc_1\ Hex_{14}\ HexNAc_{13}$	7375.6	5.58
$NeuAc_1\ Fuc_4\ Hex_{14}\ HexNAc_{14}$	7420.5	0.98
$NeuAc_2\ Fuc_6\ Hex_{13}\ HexNAc_{12}$	7435.1	1.98
$NeuAc_1\ Fuc_3\ Hex_{15}\ HexNAc_{14}$	7449.2	3.17
$NeuAc_2\ Fuc_1\ Hex_{15}\ HexNAc_{14}$	7463.4	4.52
$NeuAc_2\ Fuc_5\ Hex_{13}\ HexNAc_{13}$	7507.1	0.72
$NeuAc_1\ Fuc_6\ Hex_{14}\ HexNAc_{13}$	7521.9	1.27
$NeuAc_2\ Fuc_4\ Hex_{14}\ HexNAc_{13}$	7536.1	2.43
$NeuAc_1\ Fuc_1\ Hex_{16}\ HexNAc_{15}$	7550.5	2.96
$NeuAc_4\ Hex_{14}\ HexNAc_{13}$	7563.1	0.54
$NeuAc_1\ Fuc_5\ Hex_{14}\ HexNAc_{14}$	7594.0	0.52
$NeuAc_2\ Fuc_3\ Hex_{14}\ HexNAc_{14}$	7608.9	1.03

$NeuAc_1\ Fuc_4\ Hex_{15}\ HexNAc_{14}$	7622.9	2.38
$NeuAc_2\ Fuc_2\ Hex_{15}\ HexNAc_{14}$	7637.4	2.22
$NeuAc_3\ Hex_{15}\ HexNAc_{14}$	7650.3	1.38
$NeuAc_2\ Fuc_6\ Hex_{13}\ HexNAc_{13}$	7680.8	0.35
$NeuAc_1\ Fuc_3\ Hex_{15}\ HexNAc_{15}$	7695.8	0.96
$NeuAc_2\ Fuc_5\ Hex_{14}\ HexNAc_{13}$	7710.0	2.40
$NeuAc_1\ Fuc_2\ Hex_{16}\ HexNAc_{15}$	7723.9	1.81
$NeuAc_4\ Fuc_1\ Hex_{14}\ HexNAc_{13}$	7737.5	2.28
$NeuAc_1\ Fuc_6\ Hex_{14}\ HexNAc_{14}$	7768.1	0.35
$NeuAc_2\ Fuc_4\ Hex_{14}\ HexNAc_{14}$	7782.8	0.70
$NeuAc_1\ Fuc_5\ Hex_{15}\ HexNAc_{14}$	7797.1	1.56
$NeuAc_2\ Fuc_3\ Hex_{15}\ HexNAc_{14}$	7811.4	1.99
$NeuAc_3\ Fuc_1\ Hex_{15}\ HexNAc_{14}$	7825.1	3.18
$NeuAc_1\ Fuc_4\ Hex_{15}\ HexNAc_{15}$	7869.7	0.59
$NeuAc_2\ Fuc_6\ Hex_{14}\ HexNAc_{13}$	7884.6	1.10
$NeuAc_1\ Fuc_3\ Hex_{16}\ HexNAc_{15}$	7898.5	1.74
$NeuAc_2\ Fuc_1\ Hex_{16}\ HexNAc_{15}$	7912.8	2.45
$NeuAc_2\ Fuc_5\ Hex_{14}\ HexNAc_{14}$	7956.2	0.41
$NeuAc_1\ Fuc_6\ Hex_{15}\ HexNAc_{14}$	7971.1	0.69
$NeuAc_2\ Fuc_4\ Hex_{15}\ HexNAc_{14}$	7985.2	1.39
$NeuAc_1\ Fuc_1\ Hex_{17}\ HexNAc_{16}$	7999.9	1.57
$NeuAc_4\ Hex_{15}\ HexNAc_{14}$	8012.1	0.39
$NeuAc_1\ Fuc_5\ Hex_{15}\ HexNAc_{15}$	8043.3	0.27
$NeuAc_2\ Fuc_3\ Hex_{15}\ HexNAc_{15}$	8058.3	0.66
$NeuAc_1\ Fuc_4\ Hex_{16}\ HexNAc_{15}$	8072.0	1.51
$NeuAc_2\ Fuc_2\ Hex_{16}\ HexNAc_{15}$	8087.0	1.09
$NeuAc_3\ Hex_{16}\ HexNAc_{15}$	8100.0	0.78
$NeuAc_2\ Fuc_6\ Hex_{14}\ HexNAc_{14}$	8130.0	0.25
$NeuAc_1\ Fuc_3\ Hex_{16}\ HexNAc_{16}$	8145.0	0.60
$NeuAc_2\ Fuc_5\ Hex_{15}\ HexNAc_{14}$	8159.3	1.41
$NeuAc_1\ Fuc_2\ Hex_{17}\ HexNAc_{16}$	8173.5	0.98
$NeuAc_4\ Fuc_1\ Hex_{15}\ HexNAc_{14}$	8186.9	1.31
$NeuAc_1\ Fuc_6\ Hex_{15}\ HexNAc_{15}$	8217.4	0.22
$NeuAc_2\ Fuc_4\ Hex_{15}\ HexNAc_{15}$	8232.3	0.45
$NeuAc_1\ Fuc_5\ Hex_{16}\ HexNAc_{15}$	8246.4	0.92
$NeuAc_2\ Fuc_3\ Hex_{16}\ HexNAc_{15}$	8260.8	1.02
$NeuAc_3\ Fuc_1\ Hex_{16}\ HexNAc_{15}$	8274.5	1.78
$NeuAc_1\ Fuc_4\ Hex_{16}\ HexNAc_{16}$	8318.8	0.34
$NeuAc_2\ Fuc_6\ Hex_{15}\ HexNAc_{14}$	8333.9	0.63
$NeuAc_1\ Fuc_3\ Hex_{17}\ HexNAc_{16}$	8347.8	0.97
$NeuAc_2\ Fuc_1\ Hex_{17}\ HexNAc_{16}$	8362.2	1.39
$NeuAc_2\ Fuc_5\ Hex_{15}\ HexNAc_{15}$	8405.6	0.30
$NeuAc_1\ Fuc_6\ Hex_{16}\ HexNAc_{15}$	8420.7	0.40
$NeuAc_2\ Fuc_4\ Hex_{16}\ HexNAc_{15}$	8434.4	0.78
$NeuAc_1\ Fuc_1\ Hex_{18}\ HexNAc_{17}$	8449.4	0.80

$NeuAc_4\ Hex_{16}\ HexNAc_{15}$	8462.0	0.19
$NeuAc_1\ Fuc_5\ Hex_{16}\ HexNAc_{16}$	8492.7	0.16
$NeuAc_2\ Fuc_3\ Hex_{16}\ HexNAc_{16}$	8507.2	0.40
$NeuAc_1\ Fuc_4\ Hex_{17}\ HexNAc_{16}$	8521.1	0.87
$NeuAc_2\ Fuc_2\ Hex_{17}\ HexNAc_{16}$	8536.1	0.75
$NeuAc_3\ Hex_{17}\ HexNAc_{16}$	8549.2	0.47
$NeuAc_2\ Fuc_6\ Hex_{15}\ HexNAc_{15}$	8579.7	0.14
$NeuAc_1\ Fuc_3\ Hex_{17}\ HexNAc_{17}$	8594.0	0.36
$NeuAc_2\ Fuc_5\ Hex_{16}\ HexNAc_{15}$	8608.5	0.85
$NeuAc_1\ Fuc_2\ Hex_{18}\ HexNAc_{17}$	8623.3	0.54
$NeuAc_4\ Fuc_1\ Hex_{16}\ HexNAc_{15}$	8636.5	0.75
$NeuAc_1\ Fuc_6\ Hex_{16}\ HexNAc_{16}$	8666.7	0.17
$NeuAc_2\ Fuc_4\ Hex_{16}\ HexNAc_{16}$	8681.7	0.25
$NeuAc_1\ Fuc_5\ Hex_{17}\ HexNAc_{16}$	8696.2	0.49
$NeuAc_2\ Fuc_3\ Hex_{17}\ HexNAc_{16}$	8710.2	0.55
$NeuAc_3\ Fuc_1\ Hex_{17}\ HexNAc_{16}$	8724.1	0.98
$NeuAc_1\ Fuc_4\ Hex_{17}\ HexNAc_{17}$	8768.3	0.19
$NeuAc_2\ Fuc_6\ Hex_{16}\ HexNAc_{15}$	8783.0	0.39
$NeuAc_1\ Fuc_3\ Hex_{18}\ HexNAc_{17}$	8797.1	0.50
$NeuAc_2\ Fuc_1\ Hex_{18}\ HexNAc_{17}$	8811.7	0.67
$NeuAc_2\ Fuc_5\ Hex_{16}\ HexNAc_{16}$	8854.8	0.18
$NeuAc_1\ Fuc_6\ Hex_{17}\ HexNAc_{16}$	8869.9	0.26
$NeuAc_2\ Fuc_4\ Hex_{17}\ HexNAc_{16}$	8883.9	0.44
$NeuAc_1\ Fuc_1\ Hex_{19}\ HexNAc_{18}$	8898.9	0.44
$NeuAc_4\ Hex_{17}\ HexNAc_{16}$	8911.2	0.12
$NeuAc_1\ Fuc_5\ Hex_{17}\ HexNAc_{17}$	8942.2	0.10
$NeuAc_2\ Fuc_3\ Hex_{17}\ HexNAc_{17}$	8956.9	0.26
$NeuAc_1\ Fuc_4\ Hex_{18}\ HexNAc_{17}$	8970.3	0.55
$NeuAc_2\ Fuc_2\ Hex_{18}\ HexNAc_{17}$	8985.8	0.37
$NeuAc_3\ Hex_{18}\ HexNAc_{17}$	8998.8	0.27
$NeuAc_2\ Fuc_6\ Hex_{16}\ HexNAc_{16}$	9029.0	0.13
$NeuAc_1\ Fuc_3\ Hex_{18}\ HexNAc_{18}$	9043.5	0.24
$NeuAc_2\ Fuc_5\ Hex_{17}\ HexNAc_{16}$	9058.0	0.49
$NeuAc_1\ Fuc_2\ Hex_{19}\ HexNAc_{18}$	9072.8	0.25
$NeuAc_4\ Fuc_1\ Hex_{17}\ HexNAc_{16}$	9085.9	0.43
$NeuAc_1\ Fuc_6\ Hex_{17}\ HexNAc_{17}$	9115.8	0.11
$NeuAc_2\ Fuc_4\ Hex_{17}\ HexNAc_{17}$	9130.8	0.17
$NeuAc_1\ Fuc_5\ Hex_{18}\ HexNAc_{17}$	9145.2	0.32
$NeuAc_2\ Fuc_3\ Hex_{18}\ HexNAc_{17}$	9159.5	0.32
$NeuAc_3\ Fuc_1\ Hex_{18}\ HexNAc_{17}$	9173.7	0.55
$NeuAc_1\ Fuc_4\ Hex_{18}\ HexNAc_{18}$	9217.7	14.00
$NeuAc_2\ Fuc_6\ Hex_{17}\ HexNAc_{16}$	9232.3	0.18
$NeuAc_1\ Fuc_3\ Hex_{19}\ HexNAc_{18}$	9246.8	0.27
$NeuAc_2\ Fuc_1\ Hex_{19}\ HexNAc_{18}$	9261.1	0.36
$NeuAc_2\ Fuc_5\ Hex_{17}\ HexNAc_{17}$	9304.3	0.12

$NeuAc_1$ Fuc_6 Hex_{18} $HexNAc_{17}$	9319.2	0.14
$NeuAc_2$ Fuc_4 Hex_{18} $HexNAc_{17}$	9333.1	0.23
$NeuAc_1$ Fuc_1 Hex_{20} $HexNAc_{19}$	9348.0	0.22
$NeuAc_4$ Hex_{18} $HexNAc_{17}$	9361.5	0.08
$NeuAc_1$ Fuc_5 Hex_{18} $HexNAc_{18}$	9391.4	0.07
$NeuAc_2$ Fuc_3 Hex_{18} $HexNAc_{18}$	9406.1	0.17
$NeuAc_1$ Fuc_4 Hex_{19} $HexNAc_{18}$	9419.7	0.38
$NeuAc_2$ Fuc_2 Hex_{19} $HexNAc_{18}$	9435.1	0.15
$NeuAc_3$ Hex_{19} $HexNAc_{18}$	9448.3	0.17
$NeuAc_2$ Fuc_6 Hex_{17} $HexNAc_{17}$	9478.8	0.09
$NeuAc_1$ Fuc_3 Hex_{19} $HexNAc_{19}$	9493.4	0.14
$NeuAc_2$ Fuc_5 Hex_{18} $HexNAc_{17}$	9507.2	0.28
$NeuAc_1$ Fuc_2 Hex_{20} $HexNAc_{19}$	9521.9	0.15
$NeuAc_4$ Fuc_1 Hex_{18} $HexNAc_{17}$	9535.4	0.26
$NeuAc_1$ Fuc_6 Hex_{18} $HexNAc_{18}$	9566.0	0.07
$NeuAc_2$ Fuc_4 Hex_{18} $HexNAc_{18}$	9580.5	0.09
$NeuAc_1$ Fuc_5 Hex_{19} $HexNAc_{18}$	9595.0	0.20
$NeuAc_2$ Fuc_3 Hex_{19} $HexNAc_{18}$	9609.3	0.16
$NeuAc_3$ Fuc_1 Hex_{19} $HexNAc_{18}$	9622.7	0.33
$NeuAc_1$ Fuc_4 Hex_{19} $HexNAc_{19}$	9667.1	0.07
$NeuAc_2$ Fuc_6 Hex_{18} $HexNAc_{17}$	9681.3	0.14
$NeuAc_1$ Fuc_3 Hex_{20} $HexNAc_{19}$	9695.7	0.16
$NeuAc_2$ Fuc_1 Hex_{20} $HexNAc_{19}$	9710.3	0.21
$NeuAc_2$ Fuc_5 Hex_{18} $HexNAc_{18}$	9753.3	0.08
$NeuAc_1$ Fuc_6 Hex_{19} $HexNAc_{18}$	9768.8	0.10
$NeuAc_2$ Fuc_4 Hex_{19} $HexNAc_{18}$	9782.5	0.17
$NeuAc_1$ Fuc_1 Hex_{21} $HexNAc_{20}$	9797.8	0.15
$NeuAc_4$ Hex_{19} $HexNAc_{18}$	9811.0	0.08
$NeuAc_1$ Fuc_5 Hex_{19} $HexNAc_{19}$	9841.0	0.06
$NeuAc_2$ Fuc_3 Hex_{19} $HexNAc_{19}$	9855.6	0.09
$NeuAc_1$ Fuc_4 Hex_{20} $HexNAc_{19}$	9869.5	0.24
$NeuAc_2$ Fuc_2 Hex_{20} $HexNAc_{19}$	9884.9	0.10
$NeuAc_3$ Hex_{20} $HexNAc_{19}$	9897.9	0.13
$NeuAc_2$ Fuc_6 Hex_{18} $HexNAc_{18}$	9928.9	0.08
$NeuAc_1$ Fuc_3 Hex_{20} $HexNAc_{20}$	9943.3	0.12
$NeuAc_2$ Fuc_5 Hex_{19} $HexNAc_{18}$	9956.3	0.20
$NeuAc_1$ Fuc_2 Hex_{21} $HexNAc_{20}$	9971.1	0.09
$NeuAc_4$ Fuc_1 Hex_{19} $HexNAc_{18}$	9985.2	0.15
$NeuAc_1$ Fuc_6 Hex_{19} $HexNAc_{19}$	10015.1	0.05
$NeuAc_2$ Fuc_4 Hex_{19} $HexNAc_{19}$	10030.2	0.08
$NeuAc_1$ Fuc_5 Hex_{20} $HexNAc_{19}$	10044.1	0.12
$NeuAc_2$ Fuc_3 Hex_{20} $HexNAc_{19}$	10058.7	0.11
$NeuAc_3$ Fuc_1 Hex_{20} $HexNAc_{19}$	10072.5	0.18
$NeuAc_1$ Fuc_4 Hex_{20} $HexNAc_{20}$	10116.3	0.07
$NeuAc_2$ Fuc_6 Hex_{19} $HexNAc_{18}$	10130.8	0.08

$NeuAc_1$ Fuc_3 Hex_{21} $HexNAc_{20}$	10145.0	0.09
$NeuAc_2$ Fuc_1 Hex_{21} $HexNAc_{20}$	10160.3	0.13
$NeuAc_2$ Fuc_5 Hex_{19} $HexNAc_{19}$	10202.4	0.06
$NeuAc_1$ Fuc_6 Hex_{20} $HexNAc_{19}$	10218.4	0.08
$NeuAc_2$ Fuc_4 Hex_{20} $HexNAc_{19}$	10232.4	0.12
$NeuAc_1$ Fuc_1 Hex_{22} $HexNAc_{21}$	10247.5	0.09
$NeuAc_4$ Hex_{20} $HexNAc_{19}$	10258.8	0.03
$NeuAc_1$ Fuc_5 Hex_{20} $HexNAc_{20}$	10290.4	0.05
$NeuAc_2$ Fuc_3 Hex_{20} $HexNAc_{20}$	10304.8	0.09
$NeuAc_1$ Fuc_4 Hex_{21} $HexNAc_{20}$	10318.8	0.16
$NeuAc_2$ Fuc_2 Hex_{21} $HexNAc_{20}$	10334.6	0.07
$NeuAc_3$ Hex_{21} $HexNAc_{20}$	10346.8	0.11
$NeuAc_2$ Fuc_6 Hex_{19} $HexNAc_{19}$	10377.5	0.04
$NeuAc_1$ Fuc_3 Hex_{21} $HexNAc_{21}$	10391.8	0.07
$NeuAc_2$ Fuc_5 Hex_{20} $HexNAc_{19}$	10407.2	0.15
$NeuAc_1$ Fuc_2 Hex_{22} $HexNAc_{21}$	10421.6	0.05
$NeuAc_4$ Fuc_1 Hex_{20} $HexNAc_{19}$	10434.7	0.11
$NeuAc_1$ Fuc_6 Hex_{20} $HexNAc_{20}$	10464.1	0.05
$NeuAc_2$ Fuc_4 Hex_{20} $HexNAc_{20}$	10478.9	0.07
$NeuAc_1$ Fuc_5 Hex_{21} $HexNAc_{20}$	10493.5	0.06
$NeuAc_2$ Fuc_3 Hex_{21} $HexNAc_{20}$	10507.2	0.06
$NeuAc_3$ Fuc_1 Hex_{21} $HexNAc_{20}$	10522.5	0.12
$NeuAc_1$ Fuc_4 Hex_{21} $HexNAc_{21}$	10565.6	0.04
$NeuAc_2$ Fuc_6 Hex_{20} $HexNAc_{19}$	10580.7	0.05
$NeuAc_1$ Fuc_3 Hex_{22} $HexNAc_{21}$	10595.2	0.05
$NeuAc_2$ Fuc_1 Hex_{22} $HexNAc_{21}$	10609.3	0.10
$NeuAc_2$ Fuc_5 Hex_{20} $HexNAc_{20}$	10652.2	0.05
$NeuAc_1$ Fuc_6 Hex_{21} $HexNAc_{20}$	10667.3	0.06
$NeuAc_2$ Fuc_4 Hex_{21} $HexNAc_{20}$	10680.7	0.10
$NeuAc_1$ Fuc_1 Hex_{23} $HexNAc_{22}$	10696.5	0.05
$NeuAc_4$ Hex_{21} $HexNAc_{20}$	10709.6	0.03
$NeuAc_1$ Fuc_5 Hex_{21} $HexNAc_{21}$	10739.7	0.05
$NeuAc_2$ Fuc_3 Hex_{21} $HexNAc_{21}$	10754.9	0.05
$NeuAc_1$ Fuc_4 Hex_{22} $HexNAc_{21}$	10768.8	0.12
$NeuAc_2$ Fuc_2 Hex_{22} $HexNAc_{21}$	10783.7	0.08
$NeuAc_3$ Hex_{22} $HexNAc_{21}$	10796.0	0.08
$NeuAc_2$ Fuc_6 Hex_{20} $HexNAc_{20}$	10827.7	0.04
$NeuAc_1$ Fuc_3 Hex_{22} $HexNAc_{22}$	10841.3	0.06
$NeuAc_2$ Fuc_5 Hex_{21} $HexNAc_{20}$	10856.4	0.06
$NeuAc_1$ Fuc_2 Hex_{23} $HexNAc_{22}$	10871.2	0.03
$NeuAc_4$ Fuc_1 Hex_{21} $HexNAc_{20}$	10883.9	0.07
$NeuAc_2$ Fuc_4 Hex_{21} $HexNAc_{21}$	10929.3	0.05
$NeuAc_1$ Fuc_5 Hex_{22} $HexNAc_{21}$	10943.1	0.08
$NeuAc_2$ Fuc_3 Hex_{22} $HexNAc_{21}$	10957.2	0.07
$NeuAc_3$ Fuc_1 Hex_{22} $HexNAc_{21}$	10972.1	0.09

$NeuAc_1\ Fuc_4\ Hex_{22}\ HexNAc_{22}$	11015.5	0.03
$NeuAc_2\ Fuc_6\ Hex_{21}\ HexNAc_{20}$	11029.3	0.04
$NeuAc_1\ Fuc_3\ Hex_{23}\ HexNAc_{22}$	11044.1	0.05
$NeuAc_2\ Fuc_1\ Hex_{23}\ HexNAc_{22}$	11059.7	0.07
$NeuAc_2\ Fuc_5\ Hex_{21}\ HexNAc_{21}$	11102.0	0.04
$NeuAc_1\ Fuc_6\ Hex_{22}\ HexNAc_{21}$	11118.3	0.06
$NeuAc_2\ Fuc_4\ Hex_{22}\ HexNAc_{21}$	11131.0	0.06
$NeuAc_1\ Fuc_1\ Hex_{24}\ HexNAc_{23}$	11146.4	0.04
$NeuAc_4\ Hex_{22}\ HexNAc_{21}$	11159.4	0.04
$NeuAc_1\ Fuc_5\ Hex_{22}\ HexNAc_{22}$	11189.4	0.02
$NeuAc_2\ Fuc_3\ Hex_{22}\ HexNAc_{22}$	11203.5	0.04
$NeuAc_1\ Fuc_4\ Hex_{23}\ HexNAc_{22}$	11218.7	0.08
$NeuAc_2\ Fuc_2\ Hex_{23}\ HexNAc_{22}$	11233.8	0.05
$NeuAc_3\ Hex_{23}\ HexNAc_{22}$	11246.7	0.04
$NeuAc_2\ Fuc_6\ Hex_{21}\ HexNAc_{21}$	11275.3	0.02
$NeuAc_1\ Fuc_3\ Hex_{23}\ HexNAc_{23}$	11290.1	0.05
$NeuAc_2\ Fuc_5\ Hex_{22}\ HexNAc_{21}$	11304.3	0.06
$NeuAc_1\ Fuc_2\ Hex_{24}\ HexNAc_{23}$	11319.2	0.04
$NeuAc_4\ Fuc_1\ Hex_{22}\ HexNAc_{21}$	11332.8	0.08
$NeuAc_2\ Fuc_4\ Hex_{22}\ HexNAc_{22}$	11378.9	0.04
$NeuAc_1\ Fuc_5\ Hex_{23}\ HexNAc_{22}$	11393.0	0.04
$NeuAc_2\ Fuc_3\ Hex_{23}\ HexNAc_{22}$	11406.0	0.03
$NeuAc_3\ Fuc_1\ Hex_{23}\ HexNAc_{22}$	11420.1	0.07
$NeuAc_1\ Fuc_4\ Hex_{23}\ HexNAc_{23}$	11463.7	0.03
$NeuAc_2\ Fuc_6\ Hex_{22}\ HexNAc_{21}$	11479.0	0.04
$NeuAc_1\ Fuc_3\ Hex_{24}\ HexNAc_{23}$	11494.6	0.04
$NeuAc_2\ Fuc_1\ Hex_{24}\ HexNAc_{23}$	11508.4	0.05
$NeuAc_2\ Fuc_5\ Hex_{22}\ HexNAc_{22}$	11552.2	0.02
$NeuAc_1\ Fuc_6\ Hex_{23}\ HexNAc_{22}$	11567.1	0.03
$NeuAc_2\ Fuc_4\ Hex_{23}\ HexNAc_{22}$	11580.1	0.06
$NeuAc_1\ Fuc_1\ Hex_{25}\ HexNAc_{24}$	11595.7	0.03
$NeuAc_1\ Fuc_5\ Hex_{23}\ HexNAc_{23}$	11638.4	0.02
$NeuAc_1\ Fuc_4\ Hex_{24}\ HexNAc_{23}$	11668.8	0.04
$NeuAc_2\ Fuc_2\ Hex_{24}\ HexNAc_{23}$	11683.4	0.03
$NeuAc_3\ Hex_{24}\ HexNAc_{23}$	11694.9	0.05
$NeuAc_1\ Fuc_3\ Hex_{24}\ HexNAc_{24}$	11740.0	0.04
$NeuAc_2\ Fuc_5\ Hex_{23}\ HexNAc_{22}$	11754.0	0.05
$NeuAc_1\ Fuc_2\ Hex_{25}\ HexNAc_{24}$	11768.2	0.03
$NeuAc_4\ Fuc_1\ Hex_{23}\ HexNAc_{22}$	11782.4	0.04
$NeuAc_1\ Fuc_5\ Hex_{24}\ HexNAc_{23}$	11842.2	0.03
$NeuAc_2\ Fuc_3\ Hex_{24}\ HexNAc_{23}$	11856.2	0.03
$NeuAc_3\ Fuc_1\ Hex_{24}\ HexNAc_{23}$	11870.0	0.06
$NeuAc_1\ Fuc_3\ Hex_{25}\ HexNAc_{24}$	11942.8	0.03
$NeuAc_2\ Fuc_1\ Hex_{25}\ HexNAc_{24}$	11958.2	0.03

Percentage values of structural features (%)

**Total percentage**	263.50
**Sialylation**	
non-sialylated	62.42
total sialylated	201.18
1-sia	54.66
2-sia	124.07
3-sia	19.40
4-sia	2.97
**Fucosylation**	
non-fucosylated	166.33
total fucosylated	97.17
1-fuc	76.67
2-fuc	15.06
3-fuc	4.47
4-fuc	0.68
5-fuc	0.19
**Bisection**	
non-bisected	249.48
total bisected	14.02
sialylated, bisected	3.49
non-sialylated, bisected	10.53
fucosylated, bisected	11.22
non-fucosylated, bisected	2.80

Endo H released total N-glycan (1000-3000)

Composition	Observed monoisotopic Mass (m/z)	Relative abundance (%)
Hex_4 $HexNAc_1$	1130.5	10.75
Hex_5 $HexNAc_1$	1334.8	100.00
Hex_6 $HexNAc_1$	1538.7	93.47
Hex_5 $HexNAc_2$	1579.8	8.08
Hex_6 $HexNAc_1$ ℗	1640.7	2.03
Hex_7 $HexNAc_1$	1742.9	63.71
Hex_6 $HexNAc_2$	1783.9	5.28
Hex_5 $HexNAc_3$	1824.9	9.93
Hex_7 $HexNAc_1$ ℗	1844.8	1.04
$NeuAc_1$ Hex_5 $HexNAc_2$	1941.0	2.94
Hex_8 $HexNAc_1$	1947.0	59.28
Fuc_1 Hex_6 $HexNAc_2$	1958.0	0.55
Hex_7 $HexNAc_2$	1988.0	0.41
Fuc_1 Hex_5 $HexNAc_3$	1999.0	0.45
Hex_6 $HexNAc_3$	2029.0	5.77
$NeuAc_1$ Hex_6 $HexNAc_2$	2145.0	3.11
Hex_9 $HexNAc_1$	2151.0	46.26
$NeuAc_1$ Hex_5 $HexNAc_3$	2186.1	1.17

Fuc_1 Hex_6 $HexNAc_3$	2203.1	0.19
Hex_7 $HexNAc_3$	2233.1	0.84
$NeuAc_1$ Fuc_1 Hex_6 $HexNAc_2$	2319.1	0.08
$NeuAc_1$ Hex_7 $HexNAc_2$	2349.2	0.19
Hex_{10} $HexNAc_1$	2355.2	0.79
$NeuAc_1$ Hex_6 $HexNAc_3$	2390.2	0.55
Hex_7 $HexNAc_4$	2478.2	0.10
$NeuAc_1$ Hex_6 $HexNAc_3$	2595.3	0.15
Fuc_1 Hex_7 $HexNAc_4$	2652.3	0.02
$NeuAc_1$ Hex_6 $HexNAc_4$	2840.4	0.05

Endo H released phosphorylated N-glycan (1500-3000)

Composition	Observed monoisotopic Mass (m/z)	Relative abundance (%)
Hex_6 $HexNAc_1$ ℗	1640.9	100.00
Hex_7 $HexNAc_1$ ℗	1845.1	63.91
Hex_6 $HexNAc_2$ ℗	1886.1	4.63
Hex_6 $HexNAc_1$ ℗$_2$	1947.2	5.15
Hex_8 $HexNAc_1$ ℗	2049.2	12.70
Hex_7 $HexNAc_2$ ℗	2090.2	29.66
Hex_8 $HexNAc_1$ ℗$_2$	2151.3	8.46
Hex_7 $HexNAc_2$ ℗$_2$	2192.3	3.12
Hex_9 $HexNAc_1$ ℗	2253.3	6.00
Hex_8 $HexNAc_2$ ℗$_2$	2395.4	8.76
Hex_9 $HexNAc_3$ ℗$_2$	2844.7	5.30

Supplementary Table 2 List of assigned peaks of human lung N-glycans from GC-MS linkage analysis

RT	Assignment	Signature fragments	Relative abundance
12.34	t-Fuc	102, 115, 118, 131, 175	0.20
13.81	t-Man	102, 118, 129, 145, 161, 162, 205	0.65
14.08	t-Gal	102, 118, 129, 145, 161, 162, 205	0.41
14.94	2-Man	129, 130, 161, 190	1.00
15.21	2-Gal	129, 130, 145, 161, 174, 190, 205	0.05
15.27	3-Gal	118, 129, 234, 161, 277	0.33
15.77	6-Gal	99, 102, 118, 129, 162, 189, 233	0.51
16.11	2,4-Man	130, 190, 233	0.10
16.53	2,6-Man	129, 130, 189, 190	0.10
16.71	3,6-Man	118, 129, 189, 234	0.39
17.12	3,4,6-Man	97, 118, 139, 160, 171	0.04
17.57	t-GlcNAc	113, 117, 129, 143, 145, 159, 161, 203	0.05
18.43	4-linked GlcNAc	117, 159, 143, 233	0.81
19.27	3,4-linked GlcNAc	117, 142, 159, 172, 301	0.06
19.75	4,6-linked GlcNAc	117, 159, 261	0.11

Supplementary Table 3 List of assigned peaks of human lung O-glycans from MALDI-TOF-MS analysis

Composition	Observed Mass/Charge (m/z)	Relative abundance / %
Hex_1 $HexNAc_1$	534.3	5.10
$NeuAc_1$ Hex_1 $HexNAc_1$	896.5	65.93
Hex_2 $HexNAc_2$	983.6	3.65
$NeuAc_2$ Hex_1 $HexNAc_1$	1256.8	100.00
$NeuAc_1$ Hex_2 $HexNAc_2$	1344.9	14.33
$NeuAc_1$ Fuc_1 Hex_2 $HexNAc_2$	1519.0	1.23
$NeuAc_3$ Hex_1 $HexNAc_1$	1618.1	1.64
$NeuAc_2$ Hex_2 $HexNAc_2$	1706.1	4.08
$NeuAc_1$ Hex_3 $HexNAc_3$	1794.2	0.54
$NeuAc_1$ Fuc_1 Hex_3 $HexNAc_3$	1968.3	0.14

Supplementary Table 4 List of assigned peaks of human lung GSLs from MALDI-TOF-MS analysis

Composition	Observed Mass/Charge (m/z)	Relative abundance / %
$NeuAc_1$ Hex_1 $HexNAc_1$	854.6	100.00
Hex_3 $HexNAc_1$	942.6	60.73
Fuc_1 Hex_3 $HexNAc_1$	1116.8	7.08
$NeuAc_2$ Hex_1 $HexNAc_1$	1215.9	68.74
$NeuAc_1$ Hex_3 $HexNAc_1$	1304.0	80.28
Hex_4 $HexNAc_2$	1392.0	11.30
$NeuAc_1$ Fuc_1 Hex_3 $HexNAc_1$	1478.1	1.25
Fuc_1 Hex_4 $HexNAc_2$	1566.1	6.07
$NeuAc_2$ Hex_3 $HexNAc_1$	1665.2	1.44
$NeuAc_1$ Hex_4 $HexNAc_2$	1753.2	30.86
Hex_5 $HexNAc_3$	1841.3	6.27
$NeuAc_1$ Fuc_1 Hex_4 $HexNAc_2$	1927.4	0.40
Fuc_1 Hex_5 $HexNAc_3$	2015.4	4.80
$NeuAc_2$ Hex_4 $HexNAc_2$	2114.4	0.22
$NeuAc_1$ Hex_5 $HexNAc_3$	2202.5	11.15
Hex_6 $HexNAc_4$	2290.6	0.46
$NeuAc_1$ Fuc_1 Hex_5 $HexNAc_3$	2376.6	3.10
Fuc_1 Hex_6 $HexNAc_4$	2464.7	1.25
$NeuAc_1$ Fuc_2 Hex_5 $HexNAc_3$	2550.8	0.34
$NeuAc_2$ Hex_5 $HexNAc_3$	2563.8	0.83
Fuc_2 Hex_6 $HexNAc_4$	2638.8	1.78
$NeuAc_1$ Hex_6 $HexNAc_4$	2651.8	0.54
Hex_7 $HexNAc_5$	2739.8	0.12
Fuc_3 Hex_6 $HexNAc_4$	2812.9	0.96
$NeuAc_1$ Fuc_1 Hex_6 $HexNAc_4$	2825.9	1.64
Fuc_1 Hex_7 $HexNAc_5$	2914.1	0.06
$NeuAc_1$ Fuc_2 Hex_6 $HexNAc_4$	3000.1	0.36
Fuc_2 Hex_7 $HexNAc_5$	3088.2	0.18
$NeuAc_1$ Fuc_3 Hex_6 $HexNAc_4$	3174.3	0.02
Fuc_3 Hex_7 $HexNAc_5$	3262.3	0.22
$NeuAc_1$ Fuc_1 Hex_7 $HexNAc_5$	3275.3	0.09
$NeuAc_1$ Fuc_2 Hex_7 $HexNAc_5$	3449.5	0.50
$NeuAc_1$ Fuc_3 Hex_7 $HexNAc_5$	3623.6	0.10
Fuc_3 Hex_8 $HexNAc_6$	3711.7	0.02
$NeuAc_1$ Fuc_1 Hex_8 $HexNAc_6$	3724.7	0.01
Fuc_4 Hex_8 $HexNAc_6$	3884.8	0.01
$NeuAc_1$ Fuc_2 Hex_8 $HexNAc_6$	3899.0	0.01
$NeuAc_1$ Fuc_3 Hex_8 $HexNAc_6$	4073.1	0.01
Fuc_3 Hex_9 $HexNAc_7$	4161.1	<0.01
$NeuAc_1$ Fuc_4 Hex_8 $HexNAc_6$	4247.2	<0.01
Fuc_4 Hex_9 $HexNAc_7$	4335.3	<0.01
$NeuAc_1$ Fuc_2 Hex_9 $HexNAc_7$	4348.3	<0.01
$NeuAc_1$ Fuc_3 Hex_9 $HexNAc_7$	4522.5	<0.01
$NeuAc_1$ Fuc_4 Hex_9 $HexNAc_7$	4695.5	<0.01

Supplementary Table 5. Summary of influenza virus IHC staining results from literature

Ref	Year	First Author	Journal	Lectin α2.6-Sia	Lectin α2,3-Sia	Tissue	Preparation	SNA result	MAL result
Human Tissue									
35	2007	Nicholls	Respiratory Research	SNA	MAA-I/MAA-II	N/B/L	FFPE	+ N/B/L	+ N/B (MAA-I) +L macrophages and pneumocytes (MAA-I) +L pneumocytes (MAA-
23	2006	Shinya	Nature	SNA	MAA	L	FFPE	+ B/N/T	+ broncheoli/ alveoli
34	1993	Couceiro	Virus Research	SNA	MAA	T	FFPE	+ T	+ mucin droplets in goblet cells
36	2018	Eriksson	Scientific Reports	SNA	MAA-II	N/B/L	FFPE	+ N/B/L	+ L pneumocytes/ macrophages
Animal tissue									
33	2010	Van Poucke	Virology Journal	SNA	MAL-I/ MAL-II//MAA	B/N/L/T	FFPE/ Frozen	+ N/T/B/L	+ N/T/B glands (MAL-I), -N/T/B/L epithelial/ L glands (MAL-1), + N/T epithelial/ B
37	2000	Suzuki	Journal of Virology	SNA	MAA	T	Frozen	-	+
39	2009	Ning	Veterinary Research Comm	SNA	MAA-II	N/T/LRT	FFPE	+ T/LRT/	+ T/LRT
38	2011	Trebbien	Virology Journal	SNA	MAA-I/MAA-II	N/T/B/L	FFPE	+ N/T/B/L	+ L (MAAII)

B	Bronchus
N	Nasal
L	Lung
LRT	Lower respiratory tract
T	Trachea
+	Postive staining
-	Negative staining
